# ARHGAP9 suppresses the migration and invasion of hepatocellular carcinoma cells through up-regulating FOXJ2/E-cadherin

**DOI:** 10.1038/s41419-018-0976-0

**Published:** 2018-09-11

**Authors:** Hong Zhang, Qing-Feng Tang, Meng-Yao Sun, Chun-Yan Zhang, Jian-Yong Zhu, Yu-Li Shen, Bin Zhao, Zhi-Yi Shao, Li-Jun Zhang, Hong Zhang

**Affiliations:** 1 Central Laboratory, Seventh People’s Hospital, Shanghai University of Traditional Chinese Medicine, Shanghai, 200137 China; 2 Department of Clinical Laboratory and Central Laboratory, Putuo Hospital, Shanghai University of Traditional Chinese Medicine, Shanghai, 200062 China; 3 Department of General Surgery, Seventh People’s Hospital, Shanghai University of Traditional Chinese Medicine, Shanghai, 200137 China; 4 Institute of Interdisciplinary Medical Sciences, Shanghai University of Traditional Chinese Medicine, Shanghai, 201203 China

## Abstract

Rho GTPase activating protein 9 (ARHGAP9), a member of RhoGAP family, has been identified as a RhoGAP for Cdc42 and Rac1. Here, we aimed to clarify the expression and functional role of ARHGAP9 in hepatocellular carcinoma (HCC). By analyzing TCGA (The Cancer Genome Atlas) LIHC (liver hepatocellular carcinoma) database, we found that ARHGAP9 expression was lower in HCC tissues than in normal liver tissues, and that patients with ARHGAP9 lower expression had a significant shorter overall survival time than those with ARHGAP9 higher expression. Cell counting kit-8 (CCK-8), transwell assays and in vivo experimental lung metastasis assay revealed that ARHGAP9 overexpression could inhibit HCC cell proliferation, migration and invasion, as well as HCC lung metastases. By next-generation RNA-sequencing, we identified that a transcription factor, Forkhead Box J2 (FOXJ2), was significantly induced by ARHGAP9 overexpression in HepG2 cells. Ectopic expression of FOXJ2 in HCC cell lines also exerted inhibitory effects on cell migration and invasion. Moreover, the inhibitory effects of ARHGAP9 on HCC cell migration and invasion was significantly attenuated by FOXJ2 knockdown. Luciferase reporter assay demonstrated that ARHGAP9 enhanced the transcription of E-cadherin (CDH1) via FOXJ2. Chromatin immunoprecipitation (ChIP) assay demonstrated that FOXJ2 modulated the transcription of E-cadherin (CDH1) by directly binding to its promoter. Furthermore, Pearson’s correlation analysis indicated that the mRNA levels of ARHGAP9 in HCC tissues were positively correlated with the mRNA levels of FOXJ2 and CDH1. These data clearly show that ARHGAP9/FOXJ2 inhibit cell migration and invasion during HCC development via inducing the transcription of CDH1.

## Background

Hepatocellular carcinoma (HCC), as one of the most frequent malignancies, is the third leading cause of cancer-related deaths in the world^1,2^. More than 700,000 new cases are diagnosed each year, and more than 80% of HCCs occur in East/South-East Asia and Africa^3^. At present, it is generally believed that hepatitis B or C viral infections, alcohol-related cirrhosis and non-alcoholic steatohepatitis are main risk factors for HCC^4–6^. Despite great progress in surgical techniques, the 5-year overall survival of HCC patients remains extremely low^7^ because of late diagnosis, and high recurrence rate after surgical resection^8^. Therefore, there is an urgent need for a deeper understanding of the molecular mechanisms of HCC progression, which is helpful for the early diagnosis and novel therapeutic strategies.

Tumor metastasis, a hallmark of tumor malignancy, is a multistep, multifactorial process^9^. During the metastatic cascade, epithelial–mesenchymal transition (EMT), one dynamic cellular process, promotes the acquisition of migratory and invasive abilities and serves as a critical step^10–12^. Loss of E-cadherin has been suggested as the main characteristic of EMT^12^. Several transcription factors such as Snail1^13^, Slug^14^, Twist1, Zeb1, Zeb2^15^, Forkhead Box M1 (FOXM1)^16^, Forkhead Box Q1 (FOXQ1)^17^, and Forkhead Box J2 (FOXJ2)^18^, are known to regulate E-cadherin expression, which suggest the critical role of transcription factors in the induction of EMT.

Rho GTPase activating protein 9 (ARHGAP9), a member of RhoGAP family, consists of an amino terminal SH3 domain, followed by a WW domain, a pleckstrin homology domain, and a carboxy terminal RhoGAP domain^19^. RhoGAP proteins promote the hydrolysis of GTP-bound Rho GTPases, and thus inactivate Rho GTPases and suppress diverse cellular processes, such as gene transcription, cell cytoskeleton organization, cell proliferation, migration and invasion^20^. In vitro experiments demonstrated that ARHGAP9 has GAP activity toward Cdc42 and Rac1, and not RhoA^19^. Besides acting as a RhoGAP, ARHGAP9 can also interact with mitogen-activated protein kinases (MAPK), ERK2 and p38α, through the WW domain, and constrain the MAP kinases in their inactive states^21^. Takefuji et al. reported that the single nucleotide polymorphism of ARHGAP9 (rs11544238, Ala370Ser) is strongly associated with coronary artery spasm^22^. They found that ARHGAP9 negatively regulates cell migration of Vero cells. Sun et al. demonstrated that ARHGAP9 knockdown in gastric cancer cell line SGC7901 results in suppressed cell proliferation, migration and invasion, as well as inactivation of Akt and p38 signaling^23^. However, no studies have yet performed to define the expression of ARHGAP9 in HCC and to explore the possible role of ARHGAP9 on hepatic carcinogenesis.

In this study, we showed that ARHGAP9 expression in HCC tissues was significantly down-regulated compared with that in normal liver tissues. FOXJ2, a Forkhead Box transcription factor, was significantly induced by ARHGAP9 overexpression in HepG2 cells. FOXJ2 has been reported to repress the migration/invasion of glioma^24^, breast cancer^25^ and extrahepatic cholangiocarcinoma cells^26^. In vitro experiments demonstrated that FOXJ2 was critical for the inhibitory effects of ARHGAP9 on cell migration and invasion, and the transcription of E-cadherin. Our study provides novel insights into the tumor suppressive role of ARHGAP9 in HCC.

## Results

### Down-regulation of ARHGAP9 correlated with poor prognosis in HCC

**Fig. 1:**
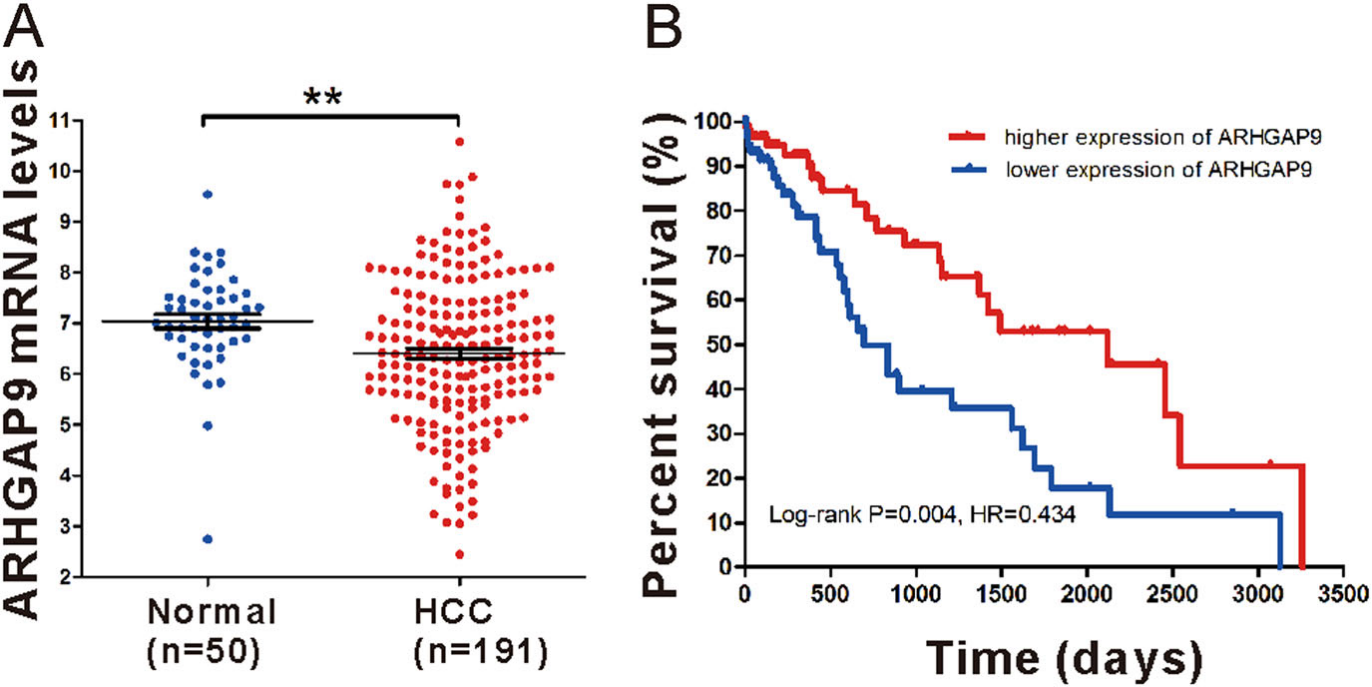
Down-regulation of ARHGAP9 indicated poor survival of HCC. **a** ARHGAP9 expression in HCC and normal liver tissues based on TCGA LIHC dataset (*P* < 0.01). **b** Survival analysis of the TCGA dataset. The overall survival time in patients with ARHGAP9 lower expression was significantly shorter than those with ARHGAP9 higher expression (*P* < 0.01)

To define ARHGAP9 expression patterns in HCC, we analyzed ARHGAP9 mRNA levels in TCGA LIHC dataset and found that ARHGAP9 mRNA was significantly lower in HCC tissues than in normal liver tissues (Fig. 1a). Further Kaplan–Meier analysis of LIHC dataset showed that patients with ARHGAP9 lower expression had a significant shorter overall survival time than those with ARHGAP9 higher expression (Fig. 1b). These data indicate the clinical value of ARHGAP9 in HCC.

### Overexpression of ARHGAP9 inhibited the proliferative, migration and invasive ability of HCC cells, as well as in vivo lung metastasis

**Fig. 2:**
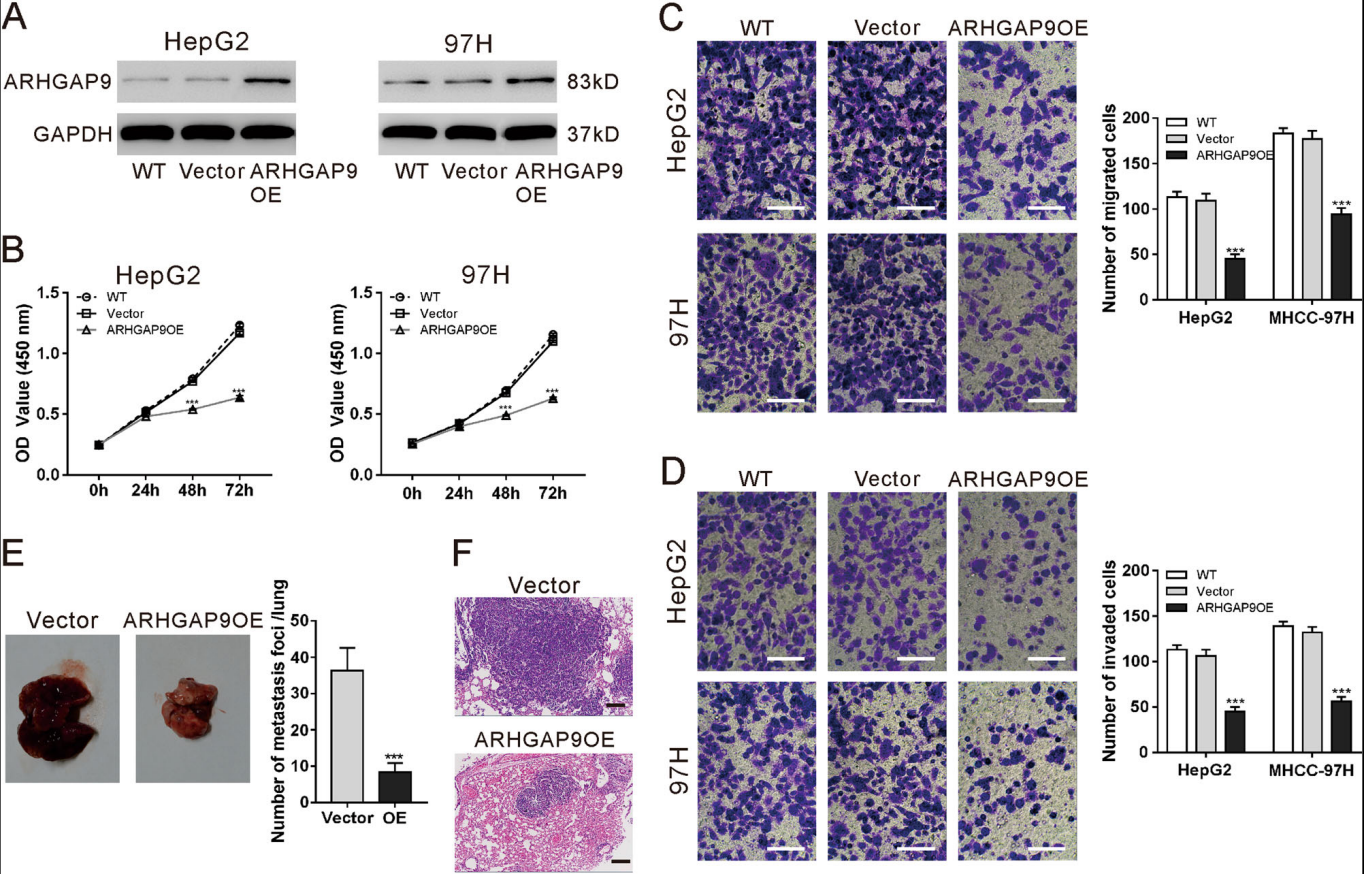
ARHGAP9 overexpression inhibited the HCC cell proliferation, migration and invasion as well as in vivo lung metastasis. **a** HepG2 and MHCC-97H cells were transduced with ARHGAP9 overexpressing (ARHGAP9OE) or control (vector) lentivirus. Cells without any treatment (WT) served as negative control. Ectopic expression of ARHGAP9 was evaluated using western blotting at 48 h after viral transduction. Representative blots from three independent experiments are shown. **b** Cell counting kit-8 (CCK-8) assays were conducted to determine cell proliferation at 0, 24, 48 and 72 h after treatment. **c**, **d** Transwell assays were conducted to determine the migration (**c**) and invasion (**d**) ability of HepG2 and MHCC-97H cells. **P < 0.01, ***P < 0.001 versus WT and vector. **e**, **f** HepG2 cells overexpressing ARHGAP9 (ARHGAP9OE) or vector (**e**) were injected intravenously into nude mice (*n* = 6 per group). The number of lung metastases was counted under a dissecting microscope and presented as mean ± SD (**f**). Lung metastases were validated by H&E staining. Scale bar: 100 μm. ***P < 0.001

To explore the role of ARHGAP9 in HCC behavior, ARHGAP9 was overexpressed in HepG2 and MHCC-97H cells by lentivirus transduction. As illustrated in Fig. 2a, ARHGAP9 protein expression was increased significantly by transduction with ARHGAP9 gene (ARHGAP9OE), while similar level was observed in wild-type (WT) cells and cells transduced with vector virus.

To examine whether ARHGAP9 affected the proliferation of HCC cells, Cell counting kit-8 (CCK-8) assay was performed in WT, vector and ARHGAP9OE cells. As illustrated in Fig. 2b, the proliferation of HepG2 and MHCC-97H cells were remarkably reduced by ARHGAP9 overexpression at 48 h and 72 h after virus transduction. To determine whether ARHGAP9 affected the migration and invasion of HCC cells, transwell assay was performed in WT, vector and ARHGAP9OE cells. Following a 24 h-culture period, HepG2 and MHCC-97H cells with ARHGAP9OE showed a significant decrease in cell migration and invasion compared to WT and vector cells (Fig. 2c, d).

To explore whether ARHGAP9 affected lung metastases of HCC, the experimental lung metastasis was produced by intravenous injection of nude mice with HepG2 cells overexpressing ARHGAP9 or vector. Compared with the vector cells, overexpression of ARHGAP9 significantly inhibited HCC lung metastases (Figs. 2e, f).

### FOXJ2 expression was elevated by ARHGAP9 overexpression

**Fig. 3:**
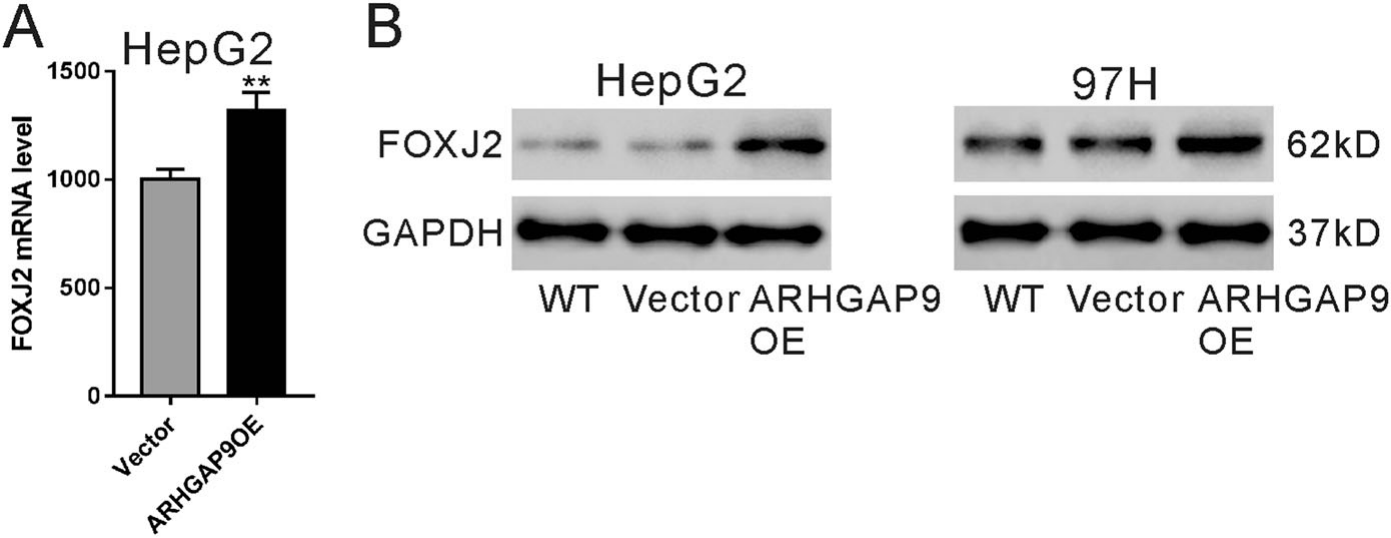
FOXJ2 expression was elevated by ARHGAP9 overexpression. **a** RNA-sequencing data showed that ARHGAP9 overexpression significantly induced mRNA level of FOXJ2 in HepG2 cells. **P < 0.01. **b** HepG2 and MHCC-97H cells were overexpressed with ARHGAP9. The protein level of FOXJ2 was significantly induced by ARHGAP9 overexpression

We then tried to explore the possible mechanisms by which ARHGAP9 suppressed HCC cell migration and invasion. By RNA sequencing, 436 (Table S1) and 342 genes (Table S2), were up-regulated and down-regulated, respectively, following ARHGAP9 overexpression, using a cut-off of *P* < 0.05 and fold change >1.2. Among these genes, a transcription factor, FOXJ2, significantly induced by ARHGAP9 overexpression in HepG2 cells (Fig. 3a) attracted our attention, because it could repress the migration/invasion of glioma^24^, breast cancer^25^ and extrahepatic cholangiocarcinoma cells^26^. Moreover, the protein levels of FOXJ2 were also increased in HepG2 and MHCC-97H cells with ARHGAP9 overexpression (Fig. 3b).

### FOXJ2 overexpression inhibited the migration and invasive ability of HCC cells

**Fig. 4:**
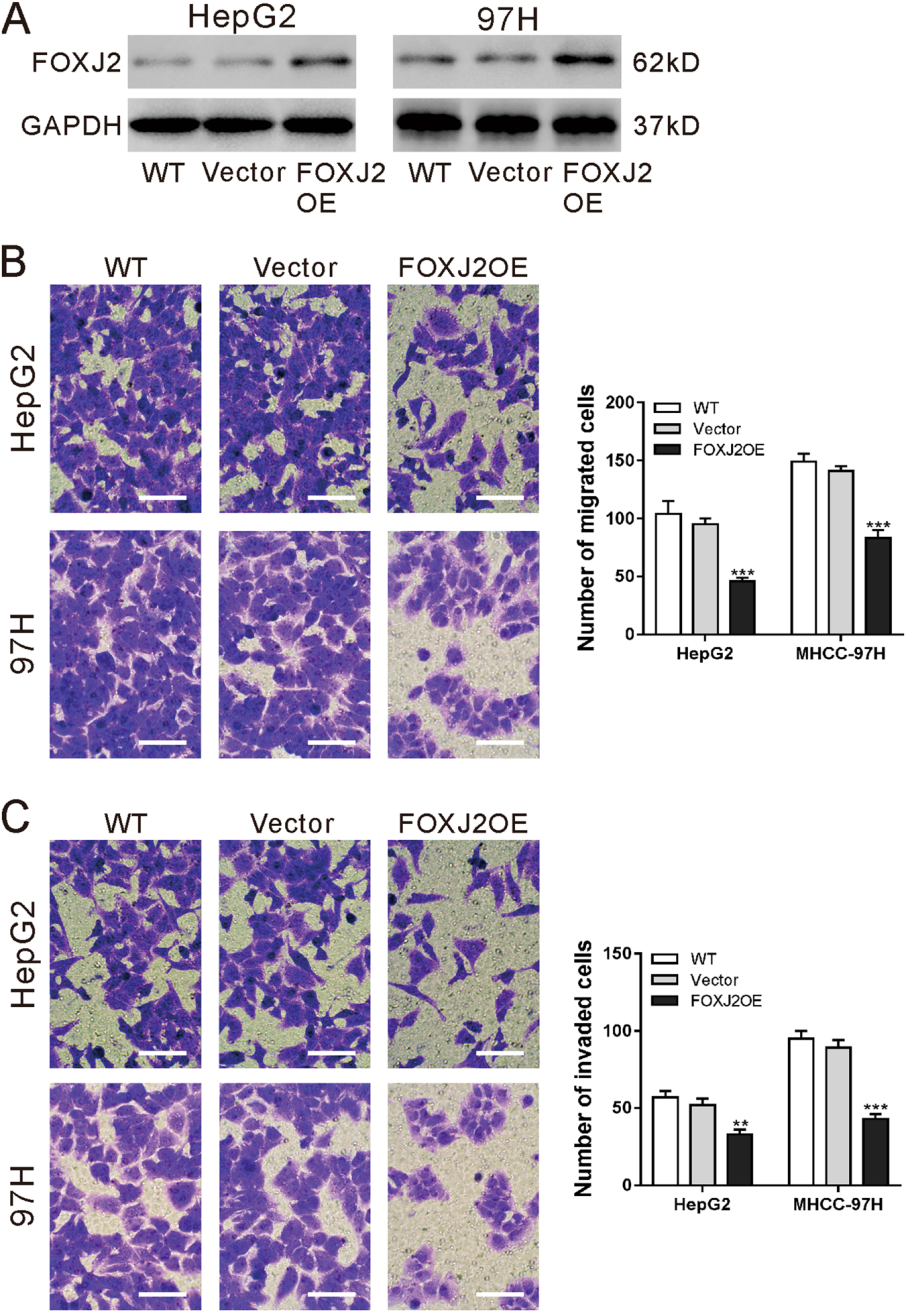
FOXJ2 overexpression inhibited the migration and invasive ability of HCC cells. **a** HepG2 and MHCC-97H cells were transduced with FOXJ2 overexpressing (FOXJ2 OE) or control (vector) lentivirus. Cells without any treatment (WT) served as negative control. Ectopic expression of FOXJ2 was evaluated using western blotting at 48 h after viral transduction. Representative blots from three independent experiments are shown. **b**, **c** Transwell assays were conducted to determine the migration (**b**) and invasion (**c**) ability of HepG2 and MHCC-97H cells. Scale bar: 100 μm. **P < 0.01, ***P < 0.001 versus WT and vector

To investigate the function of FOXJ2 in HCC cells, HepG2 and MHCC-97H cells were transduced with vector control or FOXJ2OE lentivirus. Western blot analysis confirmed the ectopic expression of FOXJ2 in both HCC cells (Fig. 4a). Transwell assays revealed that the migration and invasive ability of HepG2 and MHCC-97H cells was significantly repressed by FOXJ2 overexpression (Fig. 4b, c).

### FOXJ2 was critical for the inhibitory effects of ARHGAP9 on HCC cell migration and invasion

**Fig. 5:**
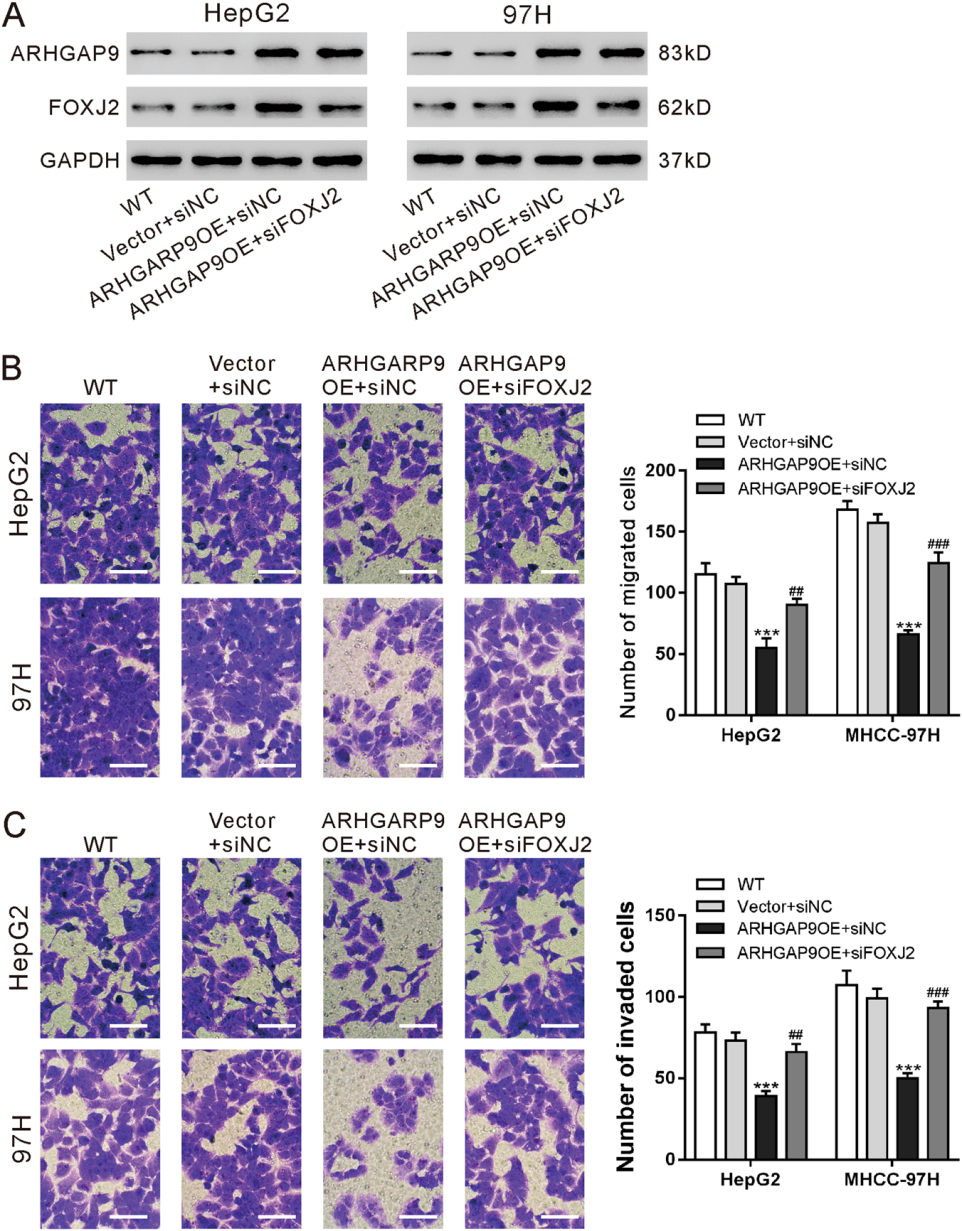
FOXJ2 was critical for the inhibitory effects of ARHGAP9 on HCC cell migration and invasion. HepG2 and MHCC-97H cells were divided into four groups: WT, cells without any treatment; vector + siNC, cells transduced with control (vector) lentivirus and transfected with control siRNA (siNC); ARHGAP9OE + siNC, cells transduced with ARHGAP9 overexpressing (ARHGAP9OE) lentivirus and transfected with control siRNA (siNC); and ARHGAP9OE + siFOXJ2, cells transduced with ARHGAP9 overexpressing (ARHGAP9OE) lentivirus and transfected with FOXJ2 siRNA (siFOXJ2). **a** Protein expression of ARHGAP9 and FOXJ2 was evaluated using western blotting at 48 h after viral transduction. Representative blots from three independent experiments are shown. **b**, **c** Transwell assays were conducted to determine the migration (**b**) and invasion (**c**) ability of HepG2 and MHCC-97H cells. Scale bar: 100 μm. ***P < 0.001 versus Vector + siNC; ^##^*P* < 0.01, ^###^*P* < 0.001 versus ARHGAP9OE + siNC

Moreover, to further explore the association between ARHGAP9 and FOXJ2, we knocked down FOXJ2 expression by siRNA transfection in HepG2 and MHCC-97H cells with ARHGAP9 overexpression (Fig. 5a). As shown in Fig. 5b, c, FOXJ2 siRNA (siFOXJ2) remarkably abrogate the inhibitory effects of ARHGAP9 overexpression on the migration and invasion of HepG2 and MHCC-97H cells.

### ARHGAP9 enhanced E-cadherin transcription via FOXJ2

**Fig. 6:**
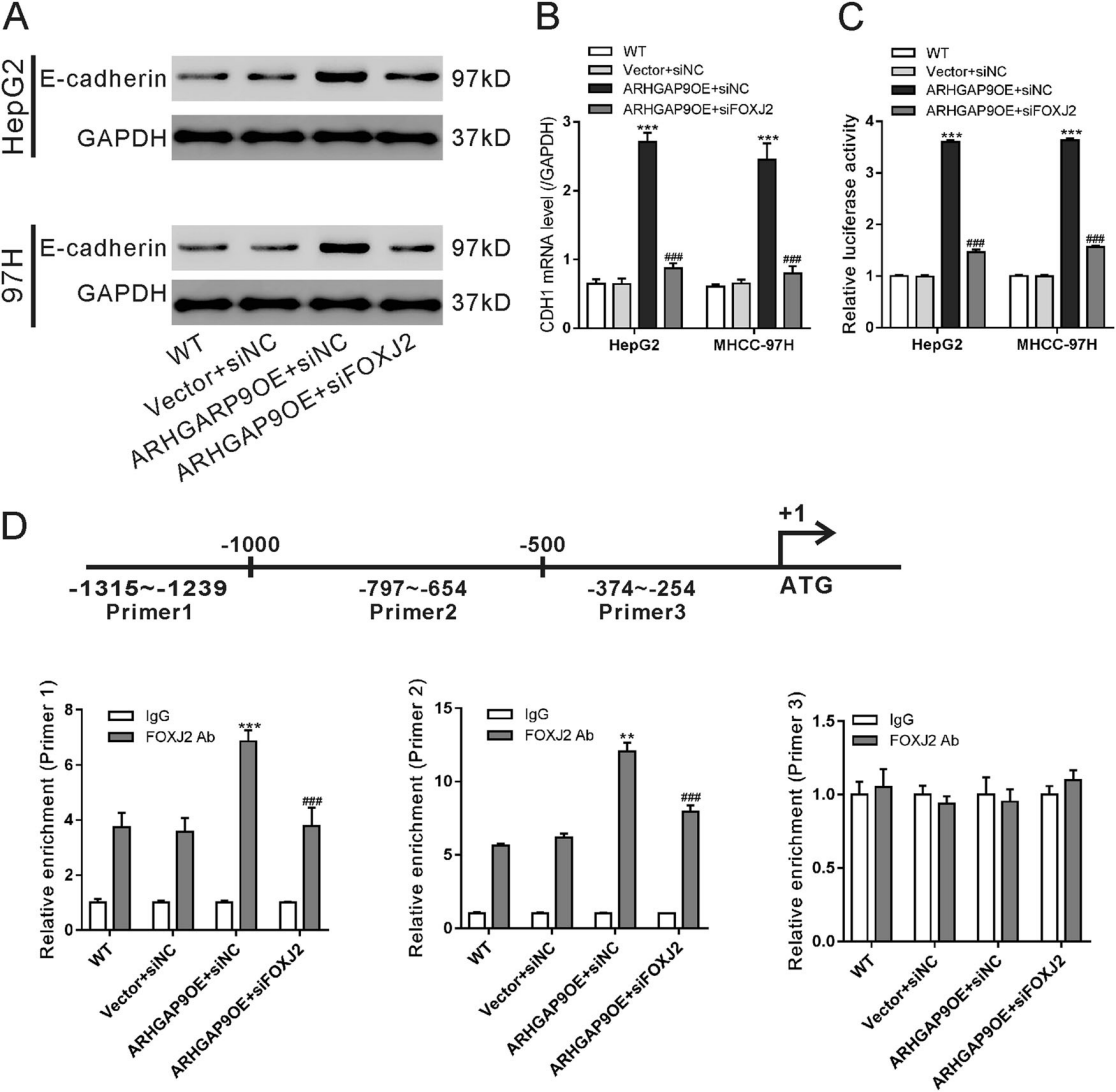
ARHGAP9 enhanced E-cadherin transcription via FOXJ2. **a**, **b** HepG2 and MHCC-97H cells were treated as described in Fig. 5. Western blot (**a**) and real-time PCR analysis (**b**) were carried out to assess the protein and mRNA expression of E-cadherin, respectively. **c** The activity of the CDH1 promoter was determined by Luciferase Reporter assays. **d** ChIP analysis was conducted in HepG2 cells to determine the binding of FOXJ2 to CDH1 promoter. IgG was a negative control. ***P < 0.001

It has been reported that FOXJ2 can regulate the expression of E-cadherin, a main factor of EMT^18^. As illustrated in Fig. 6a, b, ARHGAP9 induced the mRNA and protein levels of E-cadherin in a FOXJ2-dependent manner. Immunofluorescence staining further confirmed the changes of E-cadherin protein (Fig. S2). Luciferase assays showed that FOXJ2 knockdown significanwtly abolished ARHGAP9-induced E-cadherin promoter activity (Fig. 6c). We searched for a FOXJ2 binding site in the CDH1 promoter by using LASAGNA-Search^27^. Three binding sites were predicted, three pairs of real-time PCR primers were designed, and ChIP assays were conducted in HepG2 cells. As illustrated in Fig. 6d, FOXJ2 bound to the E-cadherin promoter regions at −1315–−1239 and −797–−654 but not to regions at −374–−254. ARHGAP9 overexpression significantly enhanced the binding of FOXJ2 to the E-cadherin promoter. FOXJ2 knockdown significantly eliminated the binding between FOXJ2 and E-cadherin promoter. These data suggest that ARHGAP9 regulates E-cadherin promoter activity via FOXJ2.

### Correlation analyses in HCC tissues

**Fig. 7:**
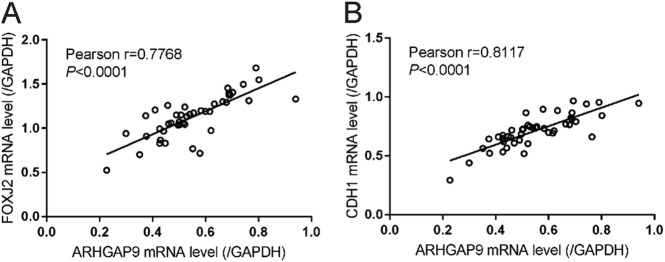
Correlation analysis in HCC tissues. Pearson’s correlation scatter plots of ARHGAP9 and FOXJ2, and of ARHGAP9 and CDH1 in HCC tissues (*n* = 45)

Real-time PCR was performed in 45 HCC tissues to assess the mRNA expression of ARHGAP9, FOXJ2 and E-cadherin (CDH1). Subsequent Pearson’s correlation analysis showed that ARHGAP9 mRNA expression was positively correlated with the expression of FOXJ2 and E-cadherin in HCC tissues (Fig. 7). These data further supported the association between ARHGA9 and FOXJ2.

## Discussion

RhoGAP proteins have been studied in different types of malignancies^28^ and the expression of several members, such as ARHGAP5^29^, DLC-1^30^ and DLC-2^31^, have been found dysregulated in HCC. However, there are few studies concerned with the expression and possible role of ARHGAP9 in HCC. Here, by analyzing TCGA LIHC dataset, we found the down-regulation of ARHGAP9 in HCC tissues, which was probably related to the prognosis of HCC patients (Fig. 1). These results implied that ARHGAP9 may be a diagnostic and prognostic marker for HCC, although more clinical data are needed to draw definitive conclusions.

We then explored the role of ARHGAP9 in HCC behavior by overexpressing ARHGAP9 in HepG2 and MHCC-97H cells. Sun et al. demonstrated that ARHGAP9 knockdown suppresses gastric cancer cell proliferation, migration and invasion^23^, while Takefuji et al. found that ARHGAP9 negatively regulates cell migration of Vero cells^22^. Here, our data of Transwell assays on HCC cell lines HepG2 and MHCC-97H (Fig. 2c, d) were consistent with the findings of Takefuji et al. The inconsistent results may be due to the different types of cells. Further in vivo lung metastasis experiments demonstrated the negative regulatory effects of ARHGAP9 in the metastases of HCC cells (Fig. 2e, f).

We also investigated the molecular mechanism how ARHGAP9 suppressed tumor metastasis. EMT is a critical step for tumor metastasis^10–12^. Several transcription factors including Forkhead Box family transcription factors (FOXM1^16^, FOXQ1^17^, FOXJ2^18^), are known to regulate the expression of E-cadherin, a main factor of EMT^12^. By RNA sequencing, we found that FOXJ2 mRNA expression was significantly increased by ARHGAP9 overexpression in HepG2 cells (Fig. 3a), and these results were validated in translational level in both HepG2 and MHCC-97H cells (Fig. 3b). In line with the findings in other types of tumor cells^24–26^, we reported here that ectopic expression of FOXJ2 remarkably repressed the migration and invasion of HCC cells (Fig. 4). FOXJ2 knockdown partially abrogated ARHGAP9-inhibted cell migration and invasion of HCC cells (Fig. 5). Moreover, our results showed that ARHGAP9 overexpression significantly induced the protein and mRNA expression, and the transcription of E-cadherin via FOXJ2 (Fig. 6a–c). Given that loss of E-cadherin is the main characteristic of EMT^12^, the increased expression of E-cadherin clearly led to reduced migratory and invasive abilities. ChIP assay demonstrated the direct binding of FOXJ2 protein to the E-cadherin (CDH1) promoter (Fig. 6d). In HCC tissues, the expression of ARHGAP9 had a positive correlation with the expression of FOXJ2 and CDH1 (Fig. 7). Accordingly, we speculated that up-regulated ARHGAP9 might increase FOXJ2 expression, which bound to the CDH1 promoter, leading to the transcription of CDH1 and the suppressed cell migration and invasion.

In conclusion, ARHGAP9 was decreased in HCC, and aberrant expression of ARHGAP9 can affect migration and invasion of HCC cells, probably through regulating FOXJ2 and its target gene CDH1. Our data identify ARHGAP9 as a potential tumor suppressor in HCC.

## Methods

### Patients and tissue samples

HCC tissues were collected from 45 patients with HCC who underwent surgical treatment at Shanghai Seventh People’s Hospital (Shanghai, China). The patients included 38 men and 7 women with a median age of 49 years. The clinical characteristics of these patients are listed in Table S3. The study protocol was approved by the Institutional Ethical Review Committee of Shanghai Seventh People’s Hospital. Written informed consent was obtained from all the participants according to the ethics committee guidelines.

### Bioinformatics analysis

ARHGAP9 mRNA levels detected in human HCC samples (*n* = 191) and normal liver tissue (*n* = 50) were obtained from TCGA (The Cancer Genome Atlas) LIHC (liver hepatocellular carcinoma) database. Student’s *t*-test was used to determine the statistical significance of ARHGAP9 expression between HCC and normal liver tissues. Kaplan–Meier survival curves were generated based on the follow-up data and analyzed by the log-rank test.

### Cell culture

The human HCC cell lines, HepG2 and MHCC-97H, were obtained from a cell bank at Chinese Academy of Sciences (Shanghai, China) and grown in Dulbecco’s modified Eagle’s medium (DMEM; Hyclone, Logan, UT, USA) containing 10% fetal bovine serum (FBS; Gibco, Grand Island, NY, USA), and 100 μg/ml streptomycin and penicillin. Both cell lines were maintained at 37 °C in a humidified atmosphere containing 5% CO_2_.

### Overexpression of ARHGAP9 and FOXJ2

The complete coding sequence (CDS) of human ARHGAP9 or FOXJ2 gene was amplified and inserted at the BamHI and EcoRI sites of pLVX-puro (Clontech, Palo Alto, CA, USA). ARHGAP9 overexpressing (ARHGAP9OE), FOXJ2 overexpressing (FOXJ2OE) and control (vector) lentivirus were prepared as described previously^32^.

### Small interfering RNA (siRNA) transfection

FOXJ2 siRNA (siFOXJ2: 5′-GCAAGCCACGAUACAGCUATT-3′) and control siRNA (siNC, 5′- CCUAAGGUUAAGUCGCCCUCG-3′) were purchased from Genepharm Technologies (Shanghai, China). Transfection was carried out using the Lipofectamine 2000 reagent (Invitrogen, Carlsbad, CA, USA) according to the manufacturer’s protocol.

### Western blot analysis

Cultured cells were lysed in ice-cold radio-immunoprecipitation assay (RIPA) lysis buffer at 4 °C for 10 min. Following centrifugation (12,000*g*) at 4 °C for 20 min, the lysates were obtained and protein concentration was determined by the BCA method. Protein samples (30 μg/lane) were then loaded onto 10% sodium dodecyl sulfate polyacrylamide gel and electroblotted onto a nitrocellulose membrane. To block the non-specific binding sites, the membranes were incubated with 5% non-fat milk (in Tris-buffered saline with 0.1% Tween-20 [TBST]) at room temperature for 60 min, and then probed with primary antibodies overnight at 4 °C. After washing three times with TBST, the membranes were incubated with the horseradish peroxidase (HRP)-conjugated secondary antibody (Beyotime, Shanghai, China) at room temperature for 1 h. Following three washes with TBST, detection was made with the enhanced chemiluminescence system (BioRad, Richmond, CA, USA). Glyceraldehyde-3′ phosphate dehydrogenase (GAPDH) was detected as loading control. The sources of primary antibodies are as follows: anti-ATHGAP9 (Ab169609) and anti-FOXJ2 (Ab22857) were from Abcam (Cambridge, MA, USA), while anti-E-cadherin (#14472) and anti-GAPDH (#5174) were from Cell Signaling Technology (Danvers, MA, USA).

### CCK-8 assay

Cell proliferation was determined using CCK-8 (Dojindo Laboratories, Japan). Cells were seeded in 96-well plates at a density of 2 × 10^3^ cells. After culture for 0, 24, 48 and 72 h, 10 μl CCK-8 reagent was added to each well and incubated at 37 °C for another 1 h. Optical density (OD) values were measured at 450 nm using Multiskan MS plate reader (Labsystems, Helsinki, Finland).

### Cell migration and invasion assays

Transwell assays were performed to determine the cell migration and invasion capacity as previously described^33^. In brief, cells were suspended in serum-free DMEM and the cell density was adjusted to approximately 2.5 × 10^5^/ml. The cell suspension was then seed to the upper chamber (Corning, Corning, NY, USA) of the inserts (0.2 ml/well), and DMEM containing 10% FBS (500 μl) was added to the lower chamber. After culturing for 24 h, the cells migrated to the bottom of the membrane were fixed with 10% formalin and stained with 0.1% crystal violet. The number of migrated cells was obtained by counting in five random fields under a light microscope. Experiments were independently repeated three times. For the invasion capacity, all the procedures were the same as described above, except that the membrane filters were pre-coated with Matrigel.

### Lung metastasis assay in vivo

The experimental protocol was approved by the Animal Experimentation Ethics Committee of Shanghai Seventh People’s Hospital. Twelve 6-week-old male BALB/c nude mice were housed under specific pathogen free (SPF) conditions. HepG2 stable cells were established by selection with puromycin (2 μg/ml; Sigma-Aldrich, St. Louis, MO, USA) for 5 days. To produce experimental lung metastasis, HepG2 stable cells (1 × 10^6^ cells) were injected intravenously via lateral tail veins. Four weeks later, the mice were sacrificed, and the number of lung metastases was counted with the use of a dissecting microscope. The collected lung tissues were fixed in 10% formalin and embedded in paraffin. Histologic examination was performed by hematoxylin and eosin (H&E) staining.

### RNA extraction and quantitative real-time PCR

Total RNA was subsequently extracted with Trizol reagent (Invitrogen) as per the manufacturer’s instructions. Experiments were independently repeated three times. cDNA synthesis kit (Thermo Fisher, Rockford, IL, USA) was used to synthesize cDNA from total RNA as per the manufacturer’s instructions. For detecting the mRNA level of ARHGAP9, FOXJ2 and E-cadherin (CDH1), quantitative real-time RT-PCR was performed on ABI 7300 system (Applied Biosystem, Foster City, CA, USA) with SYBR Green qPCR Master Mixes (Thermo Fisher). PCR procedures are as follows: incubation at 95 °C for 10 min, and 40 cycles of 95 °C for 15 s and 60 °C for 45 s. Relative mRNA expression was calculated using the 2^−ΔΔ Ct^ method^34^ with GAPDH gene as internal control. The oligonucleotides used as PCR primers were: ARHGAP9 forward: 5′- GAAGAGACCGCCCTTACAAAGC-3′; ARHGAP9 reverse: 5′-GCTCACCCGATAAATGCCATCC-3′. FOXJ2 forward: 5′-ATGGCTTCTGACCTAGAGAGTAG-3′; FOXJ2 reverse: 5′-CTGCCTCGTCTTTGCTCAGG-3′. CDH1 forward: 5′-CGAGAGCTACACGTTCACGG-3′; CDH1 reverse: 5′- GGGTGTCGAGGGAAAAATAGG-3′. GAPDH forward: 5′-CACCCACTCCTCCACCTTTG-3′; GAPDH reverse: 5′-CCACCACCCTGTTGCTGTAG-3′.

### Luciferase reporter assay

The full length CDH1 promoter was cloned into pGL3 vector (Promega, Madison, WI). HepG2 and MHCC-97H cells were co-transfected with Renilla luciferase expression vector (pRL-RSV), pGL3-CDH1 promoter and siNC (or siFOXJ2), and infected with ARHGAP9-expressing or vector lentivirus. At 48 h after transfection, the Dual Luciferase Reporter Assay System (Promega) was used to measure the activities of firefly luciferase and Renilla luciferase following the manufacturer’s instructions. The activity of firefly luciferase was normalized to that of Renilla luciferase.

### Chromatin immunoprecipitation (ChIP) assays

HepG2 cells (1 × 10^7^ cells) were treated as indicated and ChIP assays were performed at 48 h after treatment as previously described^35^. In brief, the cells were cross-linked with 1% formaldehyde at room temperature for 10 min. The cross-linked lysate was sonicated, immunoprecipitated with anti-FOXJ2 (Ab22857) or control IgG at 4 °C for 1 h, and then captured by protein A/G beads at 4 °C overnight. Quantitative real-time PCR was conducted to quantify the precipitated DNA. Primers for ChIP assays are as follows: Primer 1 forward: 5′-CTGTACAGAGCATTTATGGCTCAA-3′; Primer 1 reverse: 5′-TGTCTCCCTATGCTGTTGTGG-3′; Primer 2 forward: 5′-GAGTCTCTTGAACCCGGCA-3′; Primer 2 reverse: 5′-CCACTGAGCTAGCAGCCTAAT-3′; Primer 3 forward: 5′-CACTCCAGCTTGGGTGAAAGA-3′; Primer 3 reverse: 5′-GGCTCACTAAGACCTGGGAT-3′.

### Statistical analysis

Statistical Package for the Social Sciences software version 16.0 (SPSS, Inc., Chicago, IL, USA) was used for statistical analysis. Data are expressed as mean ± SD. Statistical significance was determined using Student’s *t*-test or one-way analysis of variance. Pearson’s correlation analysis was performed to evaluate the correlation between mRNA expression of ARHGAP9 and FOXJ2, and between ARHGAP9 and CDH1 (E-cadherin) in HCC tissues. *P-* values less than 0.05 were defined as statistical significance.

## Electronic supplementary material

Supplemental data
