# Supplementary material for: ARHGAP9 suppresses the migration and invasion of hepatocellular carcinoma cells through up-regulating FOXJ2/E-cadherin
Source: Cell Death Dis. 2018 Sep 11;9(9):916. doi: 10.1038/s41419-018-0976-0 (PMC6133947; doi:10.1038/s41419-018-0976-0)
Supplement: Supplementary file 1 — Supplemental data [file 41419_2018_976_MOESM1_ESM.docx]

**ARHGAP9 suppresses the migration and invasion of hepatocellular carcinoma cells through up-regulating FOXJ2/E-cadherin**

**Running title:** ARHGAP9 and hepatocellular carcinoma

Hong Zhang^a1^, Qing-Feng Tang^b1^, Meng-Yao Sun^b1^, Chun-Yan Zhang^a^, Jian-Yong Zhu^a^, Yu-Li Shen^a^, Bin Zhao^c^, Zhi-Yi Shao^a^, Li-Jun Zhang^a*^, Hong Zhang^a,d*^

^a^ Central Laboratory, Seventh People’s Hospital, Shanghai University of Traditional Chinese Medicine, Shanghai 200137, China

^b^ Department of Clinical Laboratory and Central Laboratory, Putuo Hospital, Shanghai University of Traditional Chinese Medicine, Shanghai 200062, China

^c^ Department of General Surgery, Seventh People’s Hospital, Shanghai University of Traditional Chinese Medicine, Shanghai 200137, China

^d^ Institute of Interdisciplinary Sciences, Shanghai University of Traditional Chinese Medicine, Shanghai 201203, China

^1^ These authors contributed equally to this work.

**Correspondence to:**

*Hong Zhang, E-mail: lkzhang97@126.com, address: 1200 Cailun Road, Shanghai 201203, China; Li-Jun Zhang, E-mail: zhanglijun0407@163.com, address: 358 Datong Road, Shanghai 200137, China.

**Materials and methods**

**RNA extraction and sequencing**

HepG2 cells were transduced with ARHGAP9 overexpressing (ARHGAP9 OE), or control (Vector) lentivirus. After 48 h, the cells were harvested by trypsinization and total RNA was subsequently extracted with Trizol reagent (Invitrogen) as per the manufacturer’s instructions. Experiments were independently repeated three times. Illumina’s TruSeq Sample Preparation Kit (Illumina, San Diego, CA) was used to prepare libraries for RNA-sequencing. Next-generation sequencing was conducted on the Illumina Genome Analyzer with the standard protocol (Illumina).

**Immunofluorescence staining**

Cells were fixed in 4% paraformaldehyde for 10 min, permeabilized with 0.5% Triton X-100 for 15 min, and blocked with 1% BSA for 1 h. Subsequently, the cells were probe with anti-E-cadherin (Abcam, ab1416) at 4°C overnight and then with Alexa Fluor 488-conjugated goat anti-mouse IgG (H+L) (Beyotime, Shanghai, China) at room temperature for 1 h. 4′, 6-diamidino-2-phenylindole (DAPI, Beyotime) was used to stain cell nuclei.

**Table S1.** Up-regulated following ARHGAP9 overexpression

|  | **Gene** | **log2FoldChange** | **lfcSE** | **stat** | **pvalue** | **padj** |
| --- | --- | --- | --- | --- | --- | --- |
| 1 | ARHGAP9 | 1.8578 | 0.4065 | -4.57 | 4.880E-06 | 2.377E-03 |
| 2 | ISG15 | 1.4583 | 0.3481 | -4.1895 | 2.800E-05 | 6.673E-03 |
| 3 | OASL | 1.4545 | 0.3169 | -4.5895 | 4.440E-06 | 2.377E-03 |
| 4 | FGFBP1 | 1.4369 | 0.3991 | -3.6006 | 3.175E-04 | 2.751E-02 |
| 5 | FASN | 1.3156 | 0.2922 | -4.5031 | 6.700E-06 | 2.733E-03 |
| 6 | CSPG5 | 1.2559 | 0.2675 | -4.6949 | 2.670E-06 | 1.961E-03 |
| 7 | PLEKHF1 | 1.2263 | 0.3571 | -3.4343 | 5.940E-04 | 4.018E-02 |
| 8 | NUDT8 | 1.2 | 0.295 | -4.0674 | 4.750E-05 | 8.472E-03 |
| 9 | CCL2 | 1.1912 | 0.3541 | -3.3641 | 7.678E-04 | 4.715E-02 |
| 10 | ZBTB42 | 1.1381 | 0.3132 | -3.6341 | 2.790E-04 | 2.451E-02 |
| 11 | EGLN3 | 1.13 | 0.3056 | -3.698 | 2.173E-04 | 2.182E-02 |
| 12 | GJC2 | 1.1189 | 0.3715 | -3.0119 | 2.596E-03 | 9.520E-02 |
| 13 | TMEM102 | 1.0678 | 0.249 | -4.2877 | 1.810E-05 | 5.311E-03 |
| 14 | RP11-567G24.1 | 1.0666 | 0.2872 | -3.7137 | 2.042E-04 | 2.101E-02 |
| 15 | SLC6A12 | 1.0664 | 0.3503 | -3.0446 | 2.330E-03 | 9.075E-02 |
| 16 | SCDP1 | 1.0575 | 0.3133 | -3.3758 | 7.361E-04 | 4.575E-02 |
| 17 | TYMP | 1.0169 | 0.391 | -2.6011 | 9.292E-03 | 1.927E-01 |
| 18 | EMP3 | 1.0166 | 0.2251 | -4.5152 | 6.330E-06 | 2.667E-03 |
| 19 | ZBTB3 | 0.9983 | 0.3307 | -3.0189 | 2.537E-03 | 9.412E-02 |
| 20 | CCL5 | 0.9869 | 0.3911 | -2.5237 | 1.161E-02 | 2.183E-01 |
| 21 | SCD | 0.9848 | 0.2583 | -3.8124 | 1.376E-04 | 1.612E-02 |
| 22 | C1QL1 | 0.9816 | 0.3464 | -2.8335 | 4.603E-03 | 1.264E-01 |
| 23 | UBASH3B | 0.9777 | 0.2299 | -4.2531 | 2.110E-05 | 5.777E-03 |
| 24 | LSS | 0.9727 | 0.2284 | -4.2588 | 2.060E-05 | 5.777E-03 |
| 25 | SOX9 | 0.9459 | 0.3468 | -2.7274 | 6.384E-03 | 1.553E-01 |
| 26 | KRT8 | 0.9456 | 0.2062 | -4.5853 | 4.530E-06 | 2.377E-03 |
| 27 | NAGS | 0.9347 | 0.33 | -2.8322 | 4.623E-03 | 1.264E-01 |
| 28 | ORAI3 | 0.9244 | 0.2912 | -3.1748 | 1.499E-03 | 7.051E-02 |
| 29 | HSPB7 | 0.9202 | 0.1858 | -4.9535 | 7.290E-07 | 7.685E-04 |
| 30 | OTX1 | 0.9133 | 0.2424 | -3.7679 | 1.646E-04 | 1.827E-02 |
| 31 | MT-ND5 | 0.9132 | 0.2377 | -3.8427 | 1.217E-04 | 1.509E-02 |
| 32 | EHF | 0.9091 | 0.3099 | -2.9339 | 3.348E-03 | 1.071E-01 |
| 33 | RARRES3 | 0.9043 | 0.3047 | -2.9678 | 3.000E-03 | 1.023E-01 |
| 34 | ATP13A2 | 0.8952 | 0.3101 | -2.8868 | 3.891E-03 | 1.168E-01 |
| 35 | SFN | 0.8941 | 0.2111 | -4.2352 | 2.280E-05 | 5.777E-03 |
| 36 | MED12 | 0.8912 | 0.2181 | -4.0857 | 4.390E-05 | 8.070E-03 |
| 37 | RPUSD2 | 0.8867 | 0.2259 | -3.9259 | 8.640E-05 | 1.234E-02 |
| 38 | PCYT2 | 0.8857 | 0.1919 | -4.6149 | 3.930E-06 | 2.377E-03 |
| 39 | TUBB4B | 0.8853 | 0.2357 | -3.7569 | 1.721E-04 | 1.876E-02 |
| 40 | hsa-mir-6723 | 0.8848 | 0.2514 | -3.5199 | 4.316E-04 | 3.434E-02 |
| 41 | KRT8P3 | 0.8793 | 0.2527 | -3.4799 | 5.015E-04 | 3.690E-02 |
| 42 | ZCCHC24 | 0.878 | 0.3029 | -2.8987 | 3.748E-03 | 1.156E-01 |
| 43 | MVK | 0.8769 | 0.192 | -4.5684 | 4.910E-06 | 2.377E-03 |
| 44 | FSTL4 | 0.8728 | 0.3403 | -2.5649 | 1.032E-02 | 2.056E-01 |
| 45 | LRFN1 | 0.8683 | 0.3197 | -2.716 | 6.607E-03 | 1.579E-01 |
| 46 | MRPL23 | 0.8553 | 0.2197 | -3.894 | 9.860E-05 | 1.321E-02 |
| 47 | ALDOC | 0.847 | 0.3412 | -2.4824 | 1.305E-02 | 2.293E-01 |
| 48 | MT-ND6 | 0.8421 | 0.2384 | -3.532 | 4.124E-04 | 3.330E-02 |
| 49 | NUDT22 | 0.8415 | 0.2952 | -2.8507 | 4.362E-03 | 1.249E-01 |
| 50 | S1PR5 | 0.841 | 0.3115 | -2.6996 | 6.941E-03 | 1.612E-01 |
| 51 | SLC25A1 | 0.8391 | 0.2972 | -2.8235 | 4.750E-03 | 1.277E-01 |
| 52 | GYS1 | 0.8352 | 0.2044 | -4.0853 | 4.400E-05 | 8.070E-03 |
| 53 | NOSIP | 0.8347 | 0.3042 | -2.7441 | 6.068E-03 | 1.510E-01 |
| 54 | ISOC2 | 0.8324 | 0.2736 | -3.0425 | 2.346E-03 | 9.075E-02 |
| 55 | ANXA6 | 0.8247 | 0.2003 | -4.1173 | 3.830E-05 | 7.950E-03 |
| 56 | RCOR2 | 0.8237 | 0.3318 | -2.4827 | 1.304E-02 | 2.293E-01 |
| 57 | TTYH3 | 0.8215 | 0.2918 | -2.8153 | 4.873E-03 | 1.300E-01 |
| 58 | APEH | 0.8193 | 0.2915 | -2.8105 | 4.946E-03 | 1.315E-01 |
| 59 | SLC38A5 | 0.8163 | 0.1367 | -5.9723 | 2.340E-09 | 8.290E-06 |
| 60 | FBXW9 | 0.8124 | 0.3078 | -2.6398 | 8.295E-03 | 1.819E-01 |
| 61 | VPS51 | 0.8117 | 0.2543 | -3.1918 | 1.414E-03 | 6.864E-02 |
| 62 | CCDC86 | 0.805 | 0.2025 | -3.9757 | 7.020E-05 | 1.073E-02 |
| 63 | ZNF574 | 0.8033 | 0.3234 | -2.4839 | 1.299E-02 | 2.293E-01 |
| 64 | EXOSC4 | 0.8029 | 0.1995 | -4.0253 | 5.690E-05 | 9.552E-03 |
| 65 | DHCR7 | 0.8029 | 0.3069 | -2.6164 | 8.887E-03 | 1.875E-01 |
| 66 | RP11-649A18.7 | 0.8007 | 0.33 | -2.4264 | 1.525E-02 | 2.492E-01 |
| 67 | FARSA | 0.8006 | 0.223 | -3.5902 | 3.304E-04 | 2.824E-02 |
| 68 | TPI1P1 | 0.7979 | 0.2058 | -3.8761 | 1.061E-04 | 1.399E-02 |
| 69 | PTMS | 0.7973 | 0.2312 | -3.4484 | 5.640E-04 | 3.896E-02 |
| 70 | TBRG4 | 0.7928 | 0.2781 | -2.8507 | 4.362E-03 | 1.249E-01 |
| 71 | TBL3 | 0.7927 | 0.2732 | -2.9014 | 3.715E-03 | 1.155E-01 |
| 72 | THEM6 | 0.7899 | 0.3156 | -2.5032 | 1.231E-02 | 2.233E-01 |
| 73 | ASL | 0.7873 | 0.2416 | -3.2593 | 1.117E-03 | 6.013E-02 |
| 74 | ALDOA | 0.7847 | 0.1945 | -4.0348 | 5.470E-05 | 9.342E-03 |
| 75 | ALKBH4 | 0.7827 | 0.2433 | -3.2166 | 1.297E-03 | 6.591E-02 |
| 76 | NPR1 | 0.7818 | 0.2607 | -2.9984 | 2.714E-03 | 9.793E-02 |
| 77 | AGFG2 | 0.7781 | 0.1952 | -3.9857 | 6.730E-05 | 1.069E-02 |
| 78 | YIF1A | 0.7775 | 0.3107 | -2.5022 | 1.234E-02 | 2.233E-01 |
| 79 | NUDT16L1 | 0.777 | 0.3083 | -2.5201 | 1.173E-02 | 2.187E-01 |
| 80 | TEAD2 | 0.7677 | 0.2016 | -3.8074 | 1.404E-04 | 1.630E-02 |
| 81 | KRT8P10 | 0.7632 | 0.2052 | -3.7188 | 2.001E-04 | 2.097E-02 |
| 82 | TUBB | 0.7625 | 0.1674 | -4.5541 | 5.260E-06 | 2.377E-03 |
| 83 | FKBPL | 0.7608 | 0.2203 | -3.4535 | 5.534E-04 | 3.868E-02 |
| 84 | TUBA1B | 0.7605 | 0.1628 | -4.6728 | 2.970E-06 | 1.978E-03 |
| 85 | CKB | 0.7601 | 0.2868 | -2.6501 | 8.048E-03 | 1.794E-01 |
| 86 | DDX60 | 0.7599 | 0.2974 | -2.5547 | 1.063E-02 | 2.084E-01 |
| 87 | PNPLA6 | 0.7595 | 0.1996 | -3.8043 | 1.422E-04 | 1.636E-02 |
| 88 | IDH2 | 0.7593 | 0.2472 | -3.0719 | 2.127E-03 | 8.708E-02 |
| 89 | G6PC3 | 0.7579 | 0.2014 | -3.7627 | 1.681E-04 | 1.849E-02 |
| 90 | MXD4 | 0.7577 | 0.1713 | -4.422 | 9.780E-06 | 3.638E-03 |
| 91 | DDX54 | 0.7572 | 0.1803 | -4.2005 | 2.660E-05 | 6.480E-03 |
| 92 | B3GNT1 | 0.7549 | 0.2223 | -3.3954 | 6.854E-04 | 4.387E-02 |
| 93 | DDN | 0.7536 | 0.2482 | -3.0357 | 2.400E-03 | 9.206E-02 |
| 94 | PIK3CD | 0.7515 | 0.2852 | -2.6353 | 8.406E-03 | 1.830E-01 |
| 95 | IP6K1 | 0.7503 | 0.1441 | -5.2074 | 1.920E-07 | 3.144E-04 |
| 96 | C17orf96 | 0.7503 | 0.2042 | -3.6733 | 2.394E-04 | 2.276E-02 |
| 97 | SRM | 0.7461 | 0.2393 | -3.1181 | 1.820E-03 | 7.966E-02 |
| 98 | SLC9A3R1 | 0.7437 | 0.2635 | -2.8225 | 4.765E-03 | 1.277E-01 |
| 99 | UBQLN4P1 | 0.7429 | 0.2619 | -2.837 | 4.554E-03 | 1.260E-01 |
| 100 | S100A10 | 0.7422 | 0.2312 | -3.2097 | 1.329E-03 | 6.651E-02 |
| 101 | GCAT | 0.7405 | 0.2635 | -2.8104 | 4.948E-03 | 1.315E-01 |
| 102 | ACLY | 0.7392 | 0.1554 | -4.7568 | 1.970E-06 | 1.555E-03 |
| 103 | TPI1 | 0.7366 | 0.2081 | -3.5389 | 4.017E-04 | 3.300E-02 |
| 104 | SLC2A1 | 0.7364 | 0.1807 | -4.0755 | 4.590E-05 | 8.299E-03 |
| 105 | TRIM21 | 0.7333 | 0.2394 | -3.0634 | 2.189E-03 | 8.830E-02 |
| 106 | TUBA1C | 0.7331 | 0.1464 | -5.0087 | 5.480E-07 | 6.933E-04 |
| 107 | OLFML2A | 0.733 | 0.2528 | -2.8999 | 3.732E-03 | 1.156E-01 |
| 108 | MT-CO1 | 0.7283 | 0.2186 | -3.3311 | 8.650E-04 | 5.078E-02 |
| 109 | SSSCA1 | 0.7283 | 0.2905 | -2.5071 | 1.217E-02 | 2.218E-01 |
| 110 | WRAP53 | 0.727 | 0.1659 | -4.3827 | 1.170E-05 | 3.802E-03 |
| 111 | DGCR6L | 0.7259 | 0.2989 | -2.4284 | 1.517E-02 | 2.488E-01 |
| 112 | RPS6KA4 | 0.7256 | 0.2196 | -3.3047 | 9.508E-04 | 5.412E-02 |
| 113 | NACC1 | 0.7241 | 0.2964 | -2.4427 | 1.458E-02 | 2.442E-01 |
| 114 | EPHX1 | 0.7231 | 0.2532 | -2.8556 | 4.295E-03 | 1.243E-01 |
| 115 | FAM63A | 0.7226 | 0.1891 | -3.8217 | 1.325E-04 | 1.581E-02 |
| 116 | DBNDD1 | 0.7211 | 0.2973 | -2.4254 | 1.529E-02 | 2.492E-01 |
| 117 | TUBBP1 | 0.7209 | 0.231 | -3.1204 | 1.806E-03 | 7.939E-02 |
| 118 | CHST12 | 0.7204 | 0.2366 | -3.0451 | 2.326E-03 | 9.075E-02 |
| 119 | ATAD3A | 0.718 | 0.2524 | -2.8451 | 4.439E-03 | 1.251E-01 |
| 120 | NR1H2 | 0.7179 | 0.2434 | -2.9494 | 3.184E-03 | 1.053E-01 |
| 121 | MT-RNR1 | 0.7177 | 0.225 | -3.1893 | 1.426E-03 | 6.864E-02 |
| 122 | MT-ND4L | 0.7175 | 0.1834 | -3.9116 | 9.170E-05 | 1.278E-02 |
| 123 | TRIM47 | 0.7173 | 0.2368 | -3.0294 | 2.450E-03 | 9.308E-02 |
| 124 | TAOK2 | 0.7163 | 0.1752 | -4.0879 | 4.350E-05 | 8.070E-03 |
| 125 | KCTD21 | 0.7135 | 0.2172 | -3.2854 | 1.018E-03 | 5.651E-02 |
| 126 | UBA1 | 0.7114 | 0.219 | -3.2484 | 1.160E-03 | 6.142E-02 |
| 127 | PRELID1 | 0.7101 | 0.2581 | -2.7515 | 5.933E-03 | 1.492E-01 |
| 128 | RASSF2 | 0.7068 | 0.2803 | -2.5216 | 1.168E-02 | 2.187E-01 |
| 129 | POP7 | 0.7064 | 0.171 | -4.1305 | 3.620E-05 | 7.631E-03 |
| 130 | ARRB1 | 0.7049 | 0.2286 | -3.0831 | 2.048E-03 | 8.497E-02 |
| 131 | TYSND1 | 0.7046 | 0.1744 | -4.0401 | 5.340E-05 | 9.257E-03 |
| 132 | TST | 0.7045 | 0.2674 | -2.6343 | 8.432E-03 | 1.833E-01 |
| 133 | CENPB | 0.7038 | 0.2489 | -2.8274 | 4.692E-03 | 1.271E-01 |
| 134 | TOMM40L | 0.7036 | 0.2276 | -3.0918 | 1.990E-03 | 8.446E-02 |
| 135 | DDB1 | 0.6996 | 0.2413 | -2.8987 | 3.747E-03 | 1.156E-01 |
| 136 | LY6K | 0.6992 | 0.2376 | -2.9423 | 3.258E-03 | 1.063E-01 |
| 137 | SF3B5 | 0.6992 | 0.2699 | -2.591 | 9.570E-03 | 1.973E-01 |
| 138 | INTS1 | 0.6986 | 0.1592 | -4.3876 | 1.150E-05 | 3.802E-03 |
| 139 | S100A4 | 0.6984 | 0.1978 | -3.5315 | 4.133E-04 | 3.330E-02 |
| 140 | RNF31 | 0.698 | 0.1952 | -3.5753 | 3.499E-04 | 2.951E-02 |
| 141 | TBXA2R | 0.6951 | 0.2036 | -3.415 | 6.379E-04 | 4.215E-02 |
| 142 | USP5 | 0.6949 | 0.1614 | -4.3059 | 1.660E-05 | 5.008E-03 |
| 143 | TRIM28 | 0.6948 | 0.1987 | -3.4969 | 4.708E-04 | 3.566E-02 |
| 144 | CDC42EP4 | 0.6942 | 0.2621 | -2.6484 | 8.086E-03 | 1.794E-01 |
| 145 | CARD6 | 0.6933 | 0.28 | -2.4758 | 1.330E-02 | 2.308E-01 |
| 146 | SPSB2 | 0.6922 | 0.2318 | -2.9861 | 2.825E-03 | 9.945E-02 |
| 147 | VARS | 0.6897 | 0.283 | -2.437 | 1.481E-02 | 2.462E-01 |
| 148 | KCNIP3 | 0.6894 | 0.2301 | -2.9954 | 2.741E-03 | 9.806E-02 |
| 149 | KRT18 | 0.6888 | 0.2031 | -3.3907 | 6.970E-04 | 4.387E-02 |
| 150 | PPP1CA | 0.6854 | 0.2184 | -3.1375 | 1.704E-03 | 7.618E-02 |
| 151 | PKM | 0.6833 | 0.1401 | -4.876 | 1.080E-06 | 9.130E-04 |
| 152 | ANO7 | 0.6819 | 0.2514 | -2.713 | 6.667E-03 | 1.579E-01 |
| 153 | HOXB13 | 0.6801 | 0.2539 | -2.6781 | 7.403E-03 | 1.697E-01 |
| 154 | ZNF865 | 0.6779 | 0.2582 | -2.6253 | 8.658E-03 | 1.856E-01 |
| 155 | RP11-289I10.2 | 0.6778 | 0.2697 | -2.5138 | 1.195E-02 | 2.200E-01 |
| 156 | FPGS | 0.6774 | 0.2624 | -2.5816 | 9.834E-03 | 1.997E-01 |
| 157 | S100A3 | 0.677 | 0.2659 | -2.5457 | 1.091E-02 | 2.103E-01 |
| 158 | FHOD1 | 0.6768 | 0.2572 | -2.6315 | 8.500E-03 | 1.834E-01 |
| 159 | CCDC167 | 0.6739 | 0.2174 | -3.1002 | 1.934E-03 | 8.292E-02 |
| 160 | CCDC85C | 0.6735 | 0.2599 | -2.5908 | 9.576E-03 | 1.973E-01 |
| 161 | RITA1 | 0.6722 | 0.2012 | -3.3406 | 8.360E-04 | 5.012E-02 |
| 162 | FKBP4 | 0.6703 | 0.2641 | -2.5375 | 1.117E-02 | 2.124E-01 |
| 163 | CALM3 | 0.669 | 0.1918 | -3.4876 | 4.873E-04 | 3.648E-02 |
| 164 | CORO1B | 0.669 | 0.2635 | -2.5391 | 1.111E-02 | 2.117E-01 |
| 165 | UBL4A | 0.6684 | 0.1663 | -4.0202 | 5.810E-05 | 9.552E-03 |
| 166 | HNF1B | 0.6659 | 0.2204 | -3.0209 | 2.520E-03 | 9.411E-02 |
| 167 | IRF2BP1 | 0.6659 | 0.2596 | -2.5652 | 1.031E-02 | 2.056E-01 |
| 168 | BRI3BP | 0.6652 | 0.2235 | -2.9757 | 2.923E-03 | 1.015E-01 |
| 169 | NT5DC2 | 0.6646 | 0.1947 | -3.4142 | 6.397E-04 | 4.215E-02 |
| 170 | SLC29A2 | 0.664 | 0.1651 | -4.0216 | 5.780E-05 | 9.552E-03 |
| 171 | HYAL1 | 0.6633 | 0.1938 | -3.4232 | 6.188E-04 | 4.142E-02 |
| 172 | ZMYM3 | 0.663 | 0.1653 | -4.0114 | 6.040E-05 | 9.791E-03 |
| 173 | VPS18 | 0.6561 | 0.2156 | -3.0435 | 2.338E-03 | 9.075E-02 |
| 174 | MCAT | 0.6561 | 0.2233 | -2.9387 | 3.295E-03 | 1.063E-01 |
| 175 | MPV17L2 | 0.6561 | 0.2562 | -2.5611 | 1.044E-02 | 2.059E-01 |
| 176 | TUFM | 0.6548 | 0.2572 | -2.5461 | 1.089E-02 | 2.103E-01 |
| 177 | MUL1 | 0.6547 | 0.1778 | -3.6816 | 2.318E-04 | 2.222E-02 |
| 178 | HRAS | 0.6541 | 0.1773 | -3.6898 | 2.244E-04 | 2.218E-02 |
| 179 | CDK9 | 0.6535 | 0.2661 | -2.4556 | 1.406E-02 | 2.380E-01 |
| 180 | TBC1D10B | 0.6529 | 0.1996 | -3.2715 | 1.070E-03 | 5.808E-02 |
| 181 | IMPDH1 | 0.6529 | 0.2337 | -2.7938 | 5.210E-03 | 1.351E-01 |
| 182 | PGAM4 | 0.6529 | 0.2389 | -2.7324 | 6.287E-03 | 1.532E-01 |
| 183 | CHPF2 | 0.6521 | 0.1652 | -3.9465 | 7.930E-05 | 1.180E-02 |
| 184 | SLC25A10 | 0.6506 | 0.2329 | -2.7939 | 5.208E-03 | 1.351E-01 |
| 185 | PUF60 | 0.6493 | 0.2398 | -2.7079 | 6.771E-03 | 1.595E-01 |
| 186 | NUP188 | 0.6489 | 0.154 | -4.2142 | 2.510E-05 | 6.216E-03 |
| 187 | QPRT | 0.6488 | 0.2275 | -2.852 | 4.345E-03 | 1.249E-01 |
| 188 | GSTM1 | 0.6459 | 0.242 | -2.6688 | 7.612E-03 | 1.723E-01 |
| 189 | CNP | 0.6453 | 0.2048 | -3.1512 | 1.626E-03 | 7.454E-02 |
| 190 | DPP3 | 0.6451 | 0.2161 | -2.9856 | 2.830E-03 | 9.945E-02 |
| 191 | GNE | 0.6442 | 0.1764 | -3.6514 | 2.609E-04 | 2.374E-02 |
| 192 | RPS6KA1 | 0.6435 | 0.2572 | -2.5023 | 1.234E-02 | 2.233E-01 |
| 193 | PRELID1P1 | 0.6433 | 0.2557 | -2.5155 | 1.188E-02 | 2.196E-01 |
| 194 | ARHGAP27 | 0.6426 | 0.2263 | -2.8393 | 4.521E-03 | 1.259E-01 |
| 195 | AAMP | 0.6421 | 0.2166 | -2.9644 | 3.033E-03 | 1.029E-01 |
| 196 | KCTD11 | 0.6411 | 0.2426 | -2.6425 | 8.230E-03 | 1.811E-01 |
| 197 | MPI | 0.639 | 0.2086 | -3.0628 | 2.193E-03 | 8.830E-02 |
| 198 | EEFSEC | 0.6374 | 0.2209 | -2.8848 | 3.916E-03 | 1.171E-01 |
| 199 | NXN | 0.6362 | 0.2228 | -2.8561 | 4.288E-03 | 1.243E-01 |
| 200 | FAM64A | 0.6352 | 0.2028 | -3.1326 | 1.733E-03 | 7.718E-02 |
| 201 | TBCD | 0.6351 | 0.1968 | -3.2267 | 1.252E-03 | 6.492E-02 |
| 202 | SYT12 | 0.6343 | 0.2348 | -2.7014 | 6.904E-03 | 1.612E-01 |
| 203 | RN7SL2 | 0.6334 | 0.2597 | -2.439 | 1.473E-02 | 2.457E-01 |
| 204 | C19orf70 | 0.6333 | 0.2323 | -2.7263 | 6.405E-03 | 1.555E-01 |
| 205 | UROD | 0.6329 | 0.214 | -2.9571 | 3.106E-03 | 1.045E-01 |
| 206 | MRPS12 | 0.6327 | 0.2502 | -2.5288 | 1.144E-02 | 2.161E-01 |
| 207 | COASY | 0.6315 | 0.242 | -2.61 | 9.055E-03 | 1.900E-01 |
| 208 | IKBKG | 0.6313 | 0.2228 | -2.8327 | 4.616E-03 | 1.264E-01 |
| 209 | EPPK1 | 0.6309 | 0.2374 | -2.6579 | 7.862E-03 | 1.767E-01 |
| 210 | EHD1 | 0.6301 | 0.2446 | -2.5767 | 9.974E-03 | 2.013E-01 |
| 211 | ATG2A | 0.6295 | 0.2054 | -3.0645 | 2.181E-03 | 8.830E-02 |
| 212 | DAB2IP | 0.6285 | 0.1678 | -3.7453 | 1.802E-04 | 1.948E-02 |
| 213 | RP11-887P2.3 | 0.6281 | 0.2375 | -2.644 | 8.192E-03 | 1.809E-01 |
| 214 | TRAPPC1 | 0.6281 | 0.2405 | -2.6119 | 9.003E-03 | 1.892E-01 |
| 215 | DUSP7 | 0.6271 | 0.2586 | -2.4246 | 1.533E-02 | 2.492E-01 |
| 216 | FLOT2 | 0.6251 | 0.2526 | -2.4742 | 1.335E-02 | 2.308E-01 |
| 217 | SLC9A3R2 | 0.6247 | 0.2236 | -2.794 | 5.206E-03 | 1.351E-01 |
| 218 | ENO1 | 0.6237 | 0.1864 | -3.3462 | 8.194E-04 | 4.936E-02 |
| 219 | TOR2A | 0.6237 | 0.2097 | -2.9736 | 2.943E-03 | 1.015E-01 |
| 220 | TADA3 | 0.6232 | 0.2473 | -2.5199 | 1.174E-02 | 2.187E-01 |
| 221 | MTX1 | 0.623 | 0.2315 | -2.6908 | 7.129E-03 | 1.646E-01 |
| 222 | PLK1 | 0.6211 | 0.1464 | -4.2416 | 2.220E-05 | 5.777E-03 |
| 223 | POR | 0.6202 | 0.2365 | -2.6223 | 8.735E-03 | 1.869E-01 |
| 224 | PSMD2 | 0.6183 | 0.1588 | -3.894 | 9.860E-05 | 1.321E-02 |
| 225 | LZTS2 | 0.6175 | 0.1938 | -3.1868 | 1.439E-03 | 6.864E-02 |
| 226 | PFKL | 0.6174 | 0.2078 | -2.9703 | 2.975E-03 | 1.017E-01 |
| 227 | KIAA0754 | 0.6173 | 0.1759 | -3.5096 | 4.488E-04 | 3.516E-02 |
| 228 | BAG6 | 0.6171 | 0.2421 | -2.5487 | 1.081E-02 | 2.100E-01 |
| 229 | CFL1 | 0.6166 | 0.2167 | -2.845 | 4.441E-03 | 1.251E-01 |
| 230 | MIEF2 | 0.6166 | 0.2179 | -2.8293 | 4.666E-03 | 1.269E-01 |
| 231 | GLUL | 0.6151 | 0.1934 | -3.1799 | 1.473E-03 | 6.980E-02 |
| 232 | EDC4 | 0.6094 | 0.1501 | -4.0605 | 4.900E-05 | 8.603E-03 |
| 233 | PSMB3 | 0.6078 | 0.1959 | -3.1031 | 1.915E-03 | 8.269E-02 |
| 234 | HMBS | 0.6077 | 0.2012 | -3.0207 | 2.522E-03 | 9.411E-02 |
| 235 | CCM2 | 0.607 | 0.2137 | -2.8403 | 4.507E-03 | 1.259E-01 |
| 236 | OPA3 | 0.6056 | 0.1597 | -3.7929 | 1.489E-04 | 1.681E-02 |
| 237 | TUBA4A | 0.6051 | 0.21 | -2.8808 | 3.966E-03 | 1.175E-01 |
| 238 | VCP | 0.6041 | 0.2081 | -2.9033 | 3.693E-03 | 1.151E-01 |
| 239 | RAVER1 | 0.6021 | 0.1868 | -3.223 | 1.269E-03 | 6.540E-02 |
| 240 | BCAR1 | 0.6004 | 0.2342 | -2.5633 | 1.037E-02 | 2.058E-01 |
| 241 | AP2S1 | 0.5989 | 0.247 | -2.4245 | 1.533E-02 | 2.492E-01 |
| 242 | LMAN2L | 0.5988 | 0.2001 | -2.9923 | 2.769E-03 | 9.867E-02 |
| 243 | AP1B1 | 0.5987 | 0.2024 | -2.9579 | 3.097E-03 | 1.045E-01 |
| 244 | KIAA0100 | 0.5985 | 0.1682 | -3.5578 | 3.740E-04 | 3.113E-02 |
| 245 | DHX37 | 0.5971 | 0.2051 | -2.9116 | 3.596E-03 | 1.126E-01 |
| 246 | KIAA0930 | 0.597 | 0.2316 | -2.5777 | 9.946E-03 | 2.013E-01 |
| 247 | PCIF1 | 0.5969 | 0.2268 | -2.6324 | 8.479E-03 | 1.834E-01 |
| 248 | RNF25 | 0.5966 | 0.1859 | -3.2096 | 1.329E-03 | 6.651E-02 |
| 249 | POLD2 | 0.5963 | 0.1758 | -3.392 | 6.940E-04 | 4.387E-02 |
| 250 | MAPK7 | 0.596 | 0.2419 | -2.4639 | 1.375E-02 | 2.346E-01 |
| 251 | SIX1 | 0.5956 | 0.2229 | -2.6718 | 7.545E-03 | 1.717E-01 |
| 252 | MT-ND4 | 0.5928 | 0.178 | -3.3304 | 8.671E-04 | 5.078E-02 |
| 253 | GCN1L1 | 0.5928 | 0.2387 | -2.4835 | 1.301E-02 | 2.293E-01 |
| 254 | FAM222B | 0.5883 | 0.1981 | -2.9703 | 2.975E-03 | 1.017E-01 |
| 255 | PPP6R1 | 0.5882 | 0.1961 | -2.9996 | 2.703E-03 | 9.793E-02 |
| 256 | ZNFX1 | 0.5876 | 0.1483 | -3.962 | 7.430E-05 | 1.119E-02 |
| 257 | PSMB6 | 0.5876 | 0.1871 | -3.14 | 1.689E-03 | 7.618E-02 |
| 258 | IMP3 | 0.5875 | 0.2366 | -2.4832 | 1.302E-02 | 2.293E-01 |
| 259 | SLC19A1 | 0.5869 | 0.2067 | -2.8388 | 4.528E-03 | 1.259E-01 |
| 260 | PTPN23 | 0.5866 | 0.1906 | -3.0775 | 2.087E-03 | 8.573E-02 |
| 261 | SLC37A4 | 0.5857 | 0.1895 | -3.0905 | 1.998E-03 | 8.455E-02 |
| 262 | GPS1 | 0.5846 | 0.2387 | -2.4486 | 1.434E-02 | 2.412E-01 |
| 263 | ACTN1 | 0.5837 | 0.222 | -2.6292 | 8.559E-03 | 1.841E-01 |
| 264 | PINK1 | 0.5836 | 0.237 | -2.4627 | 1.379E-02 | 2.347E-01 |
| 265 | THOP1 | 0.5835 | 0.1811 | -3.2223 | 1.272E-03 | 6.540E-02 |
| 266 | FDPS | 0.5833 | 0.2353 | -2.4788 | 1.318E-02 | 2.306E-01 |
| 267 | ESYT1 | 0.5825 | 0.1773 | -3.2862 | 1.016E-03 | 5.651E-02 |
| 268 | ZNF395 | 0.5796 | 0.2181 | -2.6579 | 7.863E-03 | 1.767E-01 |
| 269 | PRMT5 | 0.5792 | 0.1876 | -3.0865 | 2.025E-03 | 8.484E-02 |
| 270 | GAL | 0.5788 | 0.1876 | -3.0853 | 2.033E-03 | 8.489E-02 |
| 271 | PHB | 0.5769 | 0.138 | -4.1798 | 2.920E-05 | 6.677E-03 |
| 272 | NREP | 0.5769 | 0.2316 | -2.4907 | 1.275E-02 | 2.265E-01 |
| 273 | PRMT1 | 0.5766 | 0.2265 | -2.546 | 1.090E-02 | 2.103E-01 |
| 274 | AC016734.2 | 0.5758 | 0.2122 | -2.7133 | 6.661E-03 | 1.579E-01 |
| 275 | APOBEC3C | 0.5755 | 0.1404 | -4.0982 | 4.160E-05 | 8.070E-03 |
| 276 | UBL7 | 0.5738 | 0.1896 | -3.0267 | 2.472E-03 | 9.363E-02 |
| 277 | DTX4 | 0.5727 | 0.1765 | -3.2442 | 1.178E-03 | 6.157E-02 |
| 278 | TNNC1 | 0.5712 | 0.2217 | -2.5767 | 9.976E-03 | 2.013E-01 |
| 279 | MT-TP | 0.5704 | 0.2168 | -2.6313 | 8.506E-03 | 1.834E-01 |
| 280 | GPKOW | 0.5687 | 0.1944 | -2.9252 | 3.442E-03 | 1.094E-01 |
| 281 | TTC38 | 0.5684 | 0.1941 | -2.9291 | 3.399E-03 | 1.083E-01 |
| 282 | MPDU1 | 0.5663 | 0.2257 | -2.5095 | 1.209E-02 | 2.210E-01 |
| 283 | GPI | 0.5655 | 0.2134 | -2.6502 | 8.044E-03 | 1.794E-01 |
| 284 | AAR2 | 0.5646 | 0.2184 | -2.585 | 9.738E-03 | 1.990E-01 |
| 285 | ACACA | 0.5627 | 0.2297 | -2.4495 | 1.431E-02 | 2.410E-01 |
| 286 | SLC25A19 | 0.5626 | 0.2158 | -2.6076 | 9.117E-03 | 1.910E-01 |
| 287 | CREB3 | 0.5617 | 0.2131 | -2.6363 | 8.382E-03 | 1.830E-01 |
| 288 | PLP2 | 0.5614 | 0.1409 | -3.9847 | 6.760E-05 | 1.069E-02 |
| 289 | TNFAIP1 | 0.5613 | 0.193 | -2.9085 | 3.632E-03 | 1.134E-01 |
| 290 | EBP | 0.5604 | 0.1881 | -2.9799 | 2.883E-03 | 1.007E-01 |
| 291 | WNT10B | 0.5583 | 0.1963 | -2.8441 | 4.453E-03 | 1.252E-01 |
| 292 | UBQLN4 | 0.558 | 0.2172 | -2.5692 | 1.019E-02 | 2.038E-01 |
| 293 | PSMD13 | 0.5566 | 0.1886 | -2.9509 | 3.168E-03 | 1.052E-01 |
| 294 | HPDL | 0.5558 | 0.2018 | -2.7542 | 5.884E-03 | 1.485E-01 |
| 295 | DNAJC14 | 0.5551 | 0.1858 | -2.9876 | 2.812E-03 | 9.936E-02 |
| 296 | HCFC1 | 0.555 | 0.188 | -2.9528 | 3.150E-03 | 1.051E-01 |
| 297 | CTC-471F3.4 | 0.5537 | 0.2107 | -2.6274 | 8.604E-03 | 1.848E-01 |
| 298 | C19orf43 | 0.5527 | 0.1783 | -3.1003 | 1.933E-03 | 8.292E-02 |
| 299 | LLGL2 | 0.5515 | 0.1826 | -3.0196 | 2.531E-03 | 9.412E-02 |
| 300 | SMARCA4 | 0.5484 | 0.138 | -3.9749 | 7.040E-05 | 1.073E-02 |
| 301 | PCBP1 | 0.5479 | 0.1725 | -3.1755 | 1.496E-03 | 7.051E-02 |
| 302 | MTA2 | 0.5478 | 0.1436 | -3.8141 | 1.367E-04 | 1.612E-02 |
| 303 | MLF2 | 0.5477 | 0.2169 | -2.5254 | 1.156E-02 | 2.175E-01 |
| 304 | CELSR2 | 0.5472 | 0.1861 | -2.9409 | 3.273E-03 | 1.063E-01 |
| 305 | C12orf49 | 0.5448 | 0.1986 | -2.7423 | 6.101E-03 | 1.513E-01 |
| 306 | FAM83H | 0.543 | 0.2137 | -2.5409 | 1.106E-02 | 2.113E-01 |
| 307 | CAD | 0.5426 | 0.2089 | -2.598 | 9.378E-03 | 1.942E-01 |
| 308 | EHD2 | 0.5409 | 0.1944 | -2.7829 | 5.388E-03 | 1.388E-01 |
| 309 | WDR55 | 0.5402 | 0.1714 | -3.1514 | 1.625E-03 | 7.454E-02 |
| 310 | ABCA3 | 0.5392 | 0.2161 | -2.4953 | 1.259E-02 | 2.248E-01 |
| 311 | PPP2R5D | 0.5379 | 0.176 | -3.0558 | 2.245E-03 | 8.939E-02 |
| 312 | C11orf24 | 0.5377 | 0.1935 | -2.7789 | 5.455E-03 | 1.403E-01 |
| 313 | RGS3 | 0.5355 | 0.216 | -2.4796 | 1.315E-02 | 2.304E-01 |
| 314 | LASP1 | 0.5351 | 0.1412 | -3.7899 | 1.507E-04 | 1.687E-02 |
| 315 | PRPF8 | 0.535 | 0.1261 | -4.2419 | 2.220E-05 | 5.777E-03 |
| 316 | CAPNS1 | 0.5337 | 0.2098 | -2.5438 | 1.097E-02 | 2.109E-01 |
| 317 | MFN2 | 0.5333 | 0.1384 | -3.8538 | 1.163E-04 | 1.471E-02 |
| 318 | RAB5C | 0.5322 | 0.2089 | -2.5479 | 1.084E-02 | 2.100E-01 |
| 319 | EXOC7 | 0.532 | 0.1501 | -3.5431 | 3.955E-04 | 3.270E-02 |
| 320 | CLN3 | 0.53 | 0.1869 | -2.8365 | 4.561E-03 | 1.260E-01 |
| 321 | IPO13 | 0.5296 | 0.2179 | -2.4308 | 1.507E-02 | 2.478E-01 |
| 322 | CCNF | 0.5295 | 0.1669 | -3.1724 | 1.512E-03 | 7.058E-02 |
| 323 | FAM189B | 0.529 | 0.1835 | -2.8826 | 3.943E-03 | 1.174E-01 |
| 324 | FLRT3 | 0.5286 | 0.2134 | -2.4771 | 1.325E-02 | 2.308E-01 |
| 325 | COA4 | 0.5255 | 0.178 | -2.9517 | 3.161E-03 | 1.052E-01 |
| 326 | SPR | 0.5255 | 0.1901 | -2.7642 | 5.707E-03 | 1.454E-01 |
| 327 | PFKP | 0.5228 | 0.1778 | -2.9397 | 3.285E-03 | 1.063E-01 |
| 328 | SUFU | 0.5212 | 0.1828 | -2.8515 | 4.352E-03 | 1.249E-01 |
| 329 | ACTG1 | 0.5198 | 0.1902 | -2.7329 | 6.278E-03 | 1.532E-01 |
| 330 | RAB34 | 0.5198 | 0.204 | -2.5477 | 1.084E-02 | 2.100E-01 |
| 331 | EIF4EBP2 | 0.519 | 0.1939 | -2.6757 | 7.456E-03 | 1.706E-01 |
| 332 | DGCR14 | 0.5188 | 0.185 | -2.8042 | 5.044E-03 | 1.329E-01 |
| 333 | NSDHL | 0.5184 | 0.1714 | -3.024 | 2.495E-03 | 9.393E-02 |
| 334 | TNIP1 | 0.5182 | 0.1882 | -2.7537 | 5.893E-03 | 1.485E-01 |
| 335 | BCKDK | 0.5181 | 0.2017 | -2.569 | 1.020E-02 | 2.038E-01 |
| 336 | DCTN1 | 0.517 | 0.1915 | -2.6995 | 6.945E-03 | 1.612E-01 |
| 337 | SRRT | 0.5159 | 0.1617 | -3.1904 | 1.421E-03 | 6.864E-02 |
| 338 | HNRNPLP2 | 0.5152 | 0.1994 | -2.5844 | 9.755E-03 | 1.990E-01 |
| 339 | KIF1C | 0.5128 | 0.1865 | -2.7494 | 5.971E-03 | 1.493E-01 |
| 340 | USP22 | 0.5118 | 0.1616 | -3.1665 | 1.543E-03 | 7.149E-02 |
| 341 | PSMD3 | 0.5114 | 0.1457 | -3.5102 | 4.478E-04 | 3.516E-02 |
| 342 | LIX1L | 0.5112 | 0.173 | -2.9549 | 3.128E-03 | 1.047E-01 |
| 343 | RNF40 | 0.5108 | 0.1574 | -3.2455 | 1.173E-03 | 6.155E-02 |
| 344 | TSTA3 | 0.5104 | 0.1947 | -2.621 | 8.767E-03 | 1.869E-01 |
| 345 | MTHFR | 0.5077 | 0.1974 | -2.572 | 1.011E-02 | 2.032E-01 |
| 346 | ABCF3 | 0.5074 | 0.194 | -2.6162 | 8.893E-03 | 1.875E-01 |
| 347 | WBSCR16 | 0.5072 | 0.1936 | -2.6193 | 8.811E-03 | 1.873E-01 |
| 348 | SKIV2L | 0.5057 | 0.2022 | -2.501 | 1.239E-02 | 2.233E-01 |
| 349 | RP11-651P23.4 | 0.5053 | 0.1928 | -2.6212 | 8.763E-03 | 1.869E-01 |
| 350 | SCFD2 | 0.5036 | 0.1975 | -2.5502 | 1.077E-02 | 2.100E-01 |
| 351 | ANXA11 | 0.5017 | 0.1613 | -3.1098 | 1.872E-03 | 8.166E-02 |
| 352 | PRUNE | 0.5016 | 0.1667 | -3.0086 | 2.625E-03 | 9.569E-02 |
| 353 | RBM42 | 0.5004 | 0.1989 | -2.5164 | 1.186E-02 | 2.196E-01 |
| 354 | DHDDS | 0.5002 | 0.1735 | -2.8827 | 3.943E-03 | 1.174E-01 |
| 355 | DHRS11 | 0.4964 | 0.167 | -2.9727 | 2.952E-03 | 1.015E-01 |
| 356 | TBCB | 0.495 | 0.1932 | -2.5619 | 1.041E-02 | 2.058E-01 |
| 357 | TRIM32 | 0.4935 | 0.1983 | -2.4883 | 1.283E-02 | 2.274E-01 |
| 358 | PSMB5 | 0.4935 | 0.2035 | -2.4253 | 1.529E-02 | 2.492E-01 |
| 359 | GAPDH | 0.4932 | 0.1626 | -3.0322 | 2.428E-03 | 9.251E-02 |
| 360 | PGM1 | 0.4924 | 0.2025 | -2.4322 | 1.501E-02 | 2.478E-01 |
| 361 | GALE | 0.4916 | 0.1821 | -2.6995 | 6.945E-03 | 1.612E-01 |
| 362 | MEN1 | 0.4894 | 0.2007 | -2.4381 | 1.477E-02 | 2.458E-01 |
| 363 | USF1 | 0.489 | 0.1519 | -3.218 | 1.291E-03 | 6.585E-02 |
| 364 | TUBGCP2 | 0.4887 | 0.1891 | -2.5838 | 9.772E-03 | 1.991E-01 |
| 365 | MRPL17 | 0.4862 | 0.1494 | -3.2541 | 1.138E-03 | 6.046E-02 |
| 366 | PGD | 0.4852 | 0.1386 | -3.5015 | 4.626E-04 | 3.540E-02 |
| 367 | PPP5C | 0.4826 | 0.1553 | -3.1067 | 1.892E-03 | 8.223E-02 |
| 368 | PGP | 0.4826 | 0.1719 | -2.8073 | 4.996E-03 | 1.322E-01 |
| 369 | MECR | 0.4826 | 0.1915 | -2.5208 | 1.171E-02 | 2.187E-01 |
| 370 | PRPF6 | 0.4818 | 0.1803 | -2.6721 | 7.537E-03 | 1.717E-01 |
| 371 | FADS2 | 0.4818 | 0.184 | -2.618 | 8.845E-03 | 1.874E-01 |
| 372 | CCT3 | 0.4805 | 0.1253 | -3.8339 | 1.261E-04 | 1.549E-02 |
| 373 | AP2M1 | 0.48 | 0.1867 | -2.5717 | 1.012E-02 | 2.032E-01 |
| 374 | C14orf1 | 0.4797 | 0.1927 | -2.4889 | 1.281E-02 | 2.273E-01 |
| 375 | DHX30 | 0.4788 | 0.1683 | -2.8452 | 4.438E-03 | 1.251E-01 |
| 376 | ASF1B | 0.4764 | 0.1843 | -2.5845 | 9.751E-03 | 1.990E-01 |
| 377 | PRRC2A | 0.4759 | 0.1648 | -2.8871 | 3.888E-03 | 1.168E-01 |
| 378 | ANKRD52 | 0.4748 | 0.1953 | -2.4308 | 1.507E-02 | 2.478E-01 |
| 379 | NF2 | 0.4743 | 0.1899 | -2.4982 | 1.248E-02 | 2.233E-01 |
| 380 | EZR | 0.474 | 0.1801 | -2.6326 | 8.474E-03 | 1.834E-01 |
| 381 | TRIP6 | 0.4727 | 0.1616 | -2.9243 | 3.452E-03 | 1.094E-01 |
| 382 | ATP5G3 | 0.4717 | 0.1446 | -3.262 | 1.106E-03 | 5.980E-02 |
| 383 | ARF3 | 0.4654 | 0.1552 | -2.998 | 2.717E-03 | 9.793E-02 |
| 384 | UNC119B | 0.4641 | 0.1905 | -2.4365 | 1.483E-02 | 2.462E-01 |
| 385 | ELAC2 | 0.463 | 0.1338 | -3.4615 | 5.371E-04 | 3.797E-02 |
| 386 | CES2 | 0.4619 | 0.1784 | -2.5894 | 9.614E-03 | 1.978E-01 |
| 387 | DAXX | 0.461 | 0.1624 | -2.8394 | 4.519E-03 | 1.259E-01 |
| 388 | CHD4 | 0.4606 | 0.1329 | -3.4649 | 5.304E-04 | 3.797E-02 |
| 389 | GTF3C5 | 0.4602 | 0.1864 | -2.4688 | 1.356E-02 | 2.324E-01 |
| 390 | SH3BP5L | 0.4593 | 0.186 | -2.4692 | 1.354E-02 | 2.324E-01 |
| 391 | PSMD4 | 0.458 | 0.1445 | -3.1689 | 1.530E-03 | 7.117E-02 |
| 392 | SCAMP3 | 0.4575 | 0.1592 | -2.8738 | 4.055E-03 | 1.193E-01 |
| 393 | DHX38 | 0.4574 | 0.1421 | -3.2195 | 1.284E-03 | 6.577E-02 |
| 394 | KIAA1549 | 0.4556 | 0.1863 | -2.446 | 1.445E-02 | 2.427E-01 |
| 395 | CDK2AP1 | 0.4555 | 0.1584 | -2.8757 | 4.031E-03 | 1.189E-01 |
| 396 | TUBG1 | 0.4547 | 0.1674 | -2.7156 | 6.616E-03 | 1.579E-01 |
| 397 | DYNLL1 | 0.4525 | 0.1417 | -3.1938 | 1.404E-03 | 6.864E-02 |
| 398 | CBR1 | 0.4524 | 0.1728 | -2.6181 | 8.843E-03 | 1.874E-01 |
| 399 | FTSJ3 | 0.4516 | 0.179 | -2.5224 | 1.166E-02 | 2.187E-01 |
| 400 | PDCD11 | 0.4493 | 0.1456 | -3.0867 | 2.024E-03 | 8.484E-02 |
| 401 | MYL6 | 0.4484 | 0.1826 | -2.4551 | 1.408E-02 | 2.380E-01 |
| 402 | MAPK4 | 0.4474 | 0.1716 | -2.6068 | 9.139E-03 | 1.911E-01 |
| 403 | SLC35A4 | 0.4461 | 0.1819 | -2.453 | 1.417E-02 | 2.389E-01 |
| 404 | OGDH | 0.4382 | 0.1545 | -2.8357 | 4.573E-03 | 1.260E-01 |
| 405 | KIAA0391 | 0.4356 | 0.1674 | -2.6027 | 9.249E-03 | 1.924E-01 |
| 406 | MAGEA6 | 0.4346 | 0.1756 | -2.475 | 1.332E-02 | 2.308E-01 |
| 407 | CCT7 | 0.4283 | 0.1515 | -2.8266 | 4.704E-03 | 1.271E-01 |
| 408 | SUMO3 | 0.4282 | 0.1718 | -2.4929 | 1.267E-02 | 2.258E-01 |
| 409 | EIF3B | 0.4281 | 0.1519 | -2.8176 | 4.839E-03 | 1.294E-01 |
| 410 | HADH | 0.4251 | 0.1418 | -2.9968 | 2.729E-03 | 9.806E-02 |
| 411 | NOTCH2 | 0.4243 | 0.1307 | -3.2465 | 1.168E-03 | 6.155E-02 |
| 412 | HNRNPUL1 | 0.4236 | 0.1302 | -3.2548 | 1.135E-03 | 6.046E-02 |
| 413 | EEF2K | 0.4228 | 0.1381 | -3.0619 | 2.200E-03 | 8.830E-02 |
| 414 | COPS7A | 0.4215 | 0.1678 | -2.5122 | 1.200E-02 | 2.200E-01 |
| 415 | KDM1A | 0.4054 | 0.1306 | -3.1035 | 1.913E-03 | 8.269E-02 |
| 416 | RPUSD4 | 0.3985 | 0.1634 | -2.4387 | 1.474E-02 | 2.457E-01 |
| 417 | TNS3 | 0.3978 | 0.1628 | -2.4443 | 1.452E-02 | 2.435E-01 |
| 418 | EIF5AL1 | 0.3973 | 0.1453 | -2.7341 | 6.255E-03 | 1.530E-01 |
| 419 | EPS8L2 | 0.3962 | 0.1564 | -2.5338 | 1.128E-02 | 2.143E-01 |
| 420 | EIF4G1 | 0.3953 | 0.1416 | -2.7921 | 5.237E-03 | 1.355E-01 |
| 421 | SOGA1 | 0.3952 | 0.1369 | -2.8878 | 3.880E-03 | 1.168E-01 |
| 422 | MEA1 | 0.3894 | 0.1409 | -2.7639 | 5.711E-03 | 1.454E-01 |
| 423 | PREP | 0.3834 | 0.1556 | -2.4635 | 1.376E-02 | 2.346E-01 |
| 424 | FOXJ2 | 0.3833 | 0.1496 | -2.5624 | 1.039E-02 | 2.058E-01 |
| 425 | LAPTM4B | 0.3807 | 0.1319 | -2.8865 | 3.896E-03 | 1.168E-01 |
| 426 | PHF19 | 0.3773 | 0.1455 | -2.593 | 9.515E-03 | 1.967E-01 |
| 427 | KHSRP | 0.3736 | 0.1347 | -2.7723 | 5.566E-03 | 1.425E-01 |
| 428 | C1QBP | 0.3728 | 0.1267 | -2.9432 | 3.249E-03 | 1.063E-01 |
| 429 | TRIM14 | 0.3683 | 0.1438 | -2.5604 | 1.046E-02 | 2.060E-01 |
| 430 | AGBL5 | 0.3681 | 0.1405 | -2.6207 | 8.774E-03 | 1.869E-01 |
| 431 | SPAG7 | 0.3617 | 0.1422 | -2.5433 | 1.098E-02 | 2.109E-01 |
| 432 | POLR2H | 0.3569 | 0.1398 | -2.5532 | 1.067E-02 | 2.090E-01 |
| 433 | PIP4K2B | 0.3553 | 0.1376 | -2.5828 | 9.800E-03 | 1.993E-01 |
| 434 | HDGF | 0.3547 | 0.144 | -2.4635 | 1.376E-02 | 2.346E-01 |
| 435 | SMPD4 | 0.3541 | 0.1407 | -2.5161 | 1.187E-02 | 2.196E-01 |
| 436 | CHMP2A | 0.3522 | 0.1399 | -2.5168 | 1.184E-02 | 2.196E-01 |

**Table S2.** Down-regulated following ARHGAP9 overexpression

|  | **Gene** | **log2FoldChange** | **lfcSE** | **stat** | **pvalue** | **padj** |
| --- | --- | --- | --- | --- | --- | --- |
| 1 | AC012360.6 | -1.8297 | 0.3644 | 5.021 | 5.140E-07 | 6.933E-04 |
| 2 | DDR2 | -1.6802 | 0.3413 | 4.9236 | 8.490E-07 | 8.266E-04 |
| 3 | NR4A3 | -1.6728 | 0.3881 | 4.3104 | 1.630E-05 | 5.008E-03 |
| 4 | RND3 | -1.646 | 0.3877 | 4.2454 | 2.180E-05 | 5.777E-03 |
| 5 | SLC7A11 | -1.5574 | 0.2708 | 5.751 | 8.870E-09 | 1.970E-05 |
| 6 | SNORD63 | -1.4264 | 0.3489 | 4.0884 | 4.340E-05 | 8.070E-03 |
| 7 | PPP1R15A | -1.3679 | 0.3054 | 4.4796 | 7.480E-06 | 2.956E-03 |
| 8 | ATF3 | -1.3387 | 0.401 | 3.3383 | 8.428E-04 | 5.029E-02 |
| 9 | RP11-845M18.6 | -1.3232 | 0.39 | 3.3924 | 6.927E-04 | 4.387E-02 |
| 10 | CXCL2 | -1.3057 | 0.4098 | 3.1862 | 1.442E-03 | 6.864E-02 |
| 11 | GPT | -1.3013 | 0.3778 | 3.4447 | 5.717E-04 | 3.896E-02 |
| 12 | NR4A1 | -1.2902 | 0.3801 | 3.3941 | 6.884E-04 | 4.387E-02 |
| 13 | CEP290 | -1.2828 | 0.2155 | 5.9537 | 2.620E-09 | 8.290E-06 |
| 14 | ADM2 | -1.2805 | 0.4 | 3.2016 | 1.367E-03 | 6.754E-02 |
| 15 | CYP27B1 | -1.2755 | 0.2602 | 4.9025 | 9.460E-07 | 8.549E-04 |
| 16 | IGFN1 | -1.2451 | 0.3763 | 3.3087 | 9.372E-04 | 5.389E-02 |
| 17 | CTC-459F4.1 | -1.2411 | 0.378 | 3.2829 | 1.027E-03 | 5.659E-02 |
| 18 | NEAT1 | -1.234 | 0.2373 | 5.2004 | 1.990E-07 | 3.144E-04 |
| 19 | THBS1 | -1.1831 | 0.388 | 3.0489 | 2.297E-03 | 9.064E-02 |
| 20 | ULBP1 | -1.1788 | 0.3751 | 3.1426 | 1.674E-03 | 7.592E-02 |
| 21 | ANKRD1 | -1.1744 | 0.4059 | 2.8931 | 3.815E-03 | 1.166E-01 |
| 22 | AC137932.1 | -1.1641 | 0.3007 | 3.8716 | 1.081E-04 | 1.410E-02 |
| 23 | TNFAIP3 | -1.1612 | 0.352 | 3.299 | 9.702E-04 | 5.479E-02 |
| 24 | SAPCD1 | -1.1555 | 0.3956 | 2.921 | 3.489E-03 | 1.101E-01 |
| 25 | RP11-54O7.1 | -1.1531 | 0.3774 | 3.0555 | 2.247E-03 | 8.939E-02 |
| 26 | C5AR1 | -1.1531 | 0.3866 | 2.9827 | 2.857E-03 | 1.001E-01 |
| 27 | ZNF695 | -1.1315 | 0.3053 | 3.7066 | 2.100E-04 | 2.126E-02 |
| 28 | CENPE | -1.1273 | 0.2835 | 3.9762 | 7.000E-05 | 1.073E-02 |
| 29 | AP001505.9 | -1.1197 | 0.2683 | 4.1738 | 3.000E-05 | 6.677E-03 |
| 30 | PABPC1L | -1.0865 | 0.1745 | 6.2283 | 4.710E-10 | 2.980E-06 |
| 31 | NFKBIZ | -1.0772 | 0.2754 | 3.9109 | 9.200E-05 | 1.278E-02 |
| 32 | ANKRD12 | -1.0745 | 0.2598 | 4.1362 | 3.530E-05 | 7.570E-03 |
| 33 | GOLGA4 | -1.0593 | 0.1633 | 6.4881 | 8.700E-11 | 1.100E-06 |
| 34 | RP11-30L15.4 | -1.0551 | 0.3426 | 3.0794 | 2.074E-03 | 8.546E-02 |
| 35 | TAS2R14 | -1.0544 | 0.2985 | 3.5324 | 4.118E-04 | 3.330E-02 |
| 36 | TXNDC5 | -1.0531 | 0.2752 | 3.8262 | 1.301E-04 | 1.568E-02 |
| 37 | TRIML2 | -1.0528 | 0.3181 | 3.3093 | 9.352E-04 | 5.389E-02 |
| 38 | STC2 | -1.0503 | 0.3677 | 2.8566 | 4.281E-03 | 1.243E-01 |
| 39 | AMOTL2 | -1.0489 | 0.4026 | 2.6052 | 9.182E-03 | 1.917E-01 |
| 40 | FLNB-AS1 | -1.044 | 0.3023 | 3.4536 | 5.532E-04 | 3.868E-02 |
| 41 | CDKN2AIP | -1.044 | 0.3183 | 3.2799 | 1.038E-03 | 5.662E-02 |
| 42 | EID3 | -1.0353 | 0.3005 | 3.4446 | 5.718E-04 | 3.896E-02 |
| 43 | CEBPG | -1.0257 | 0.2797 | 3.6673 | 2.451E-04 | 2.276E-02 |
| 44 | ASMTL-AS1 | -1.0169 | 0.3205 | 3.1727 | 1.510E-03 | 7.058E-02 |
| 45 | CYR61 | -1.0157 | 0.4125 | 2.4622 | 1.381E-02 | 2.348E-01 |
| 46 | EXOC3L4 | -1.007 | 0.3222 | 3.1255 | 1.775E-03 | 7.879E-02 |
| 47 | C8orf4 | -1.0004 | 0.3411 | 2.9333 | 3.354E-03 | 1.071E-01 |
| 48 | GOLGA6L20 | -0.9981 | 0.3133 | 3.1859 | 1.443E-03 | 6.864E-02 |
| 49 | CTC-308K20.1 | -0.9962 | 0.3657 | 2.7242 | 6.445E-03 | 1.559E-01 |
| 50 | SLFN5 | -0.9841 | 0.2519 | 3.906 | 9.380E-05 | 1.290E-02 |
| 51 | ROR1 | -0.983 | 0.3182 | 3.0889 | 2.009E-03 | 8.471E-02 |
| 52 | FRA10AC1 | -0.9797 | 0.2132 | 4.596 | 4.310E-06 | 2.377E-03 |
| 53 | NEIL1 | -0.9794 | 0.376 | 2.6045 | 9.200E-03 | 1.917E-01 |
| 54 | LINC00174 | -0.9791 | 0.332 | 2.949 | 3.188E-03 | 1.053E-01 |
| 55 | NPIPB15 | -0.9755 | 0.2506 | 3.8925 | 9.920E-05 | 1.321E-02 |
| 56 | TTC14 | -0.963 | 0.2183 | 4.411 | 1.030E-05 | 3.719E-03 |
| 57 | DLGAP1-AS2 | -0.9618 | 0.2765 | 3.4783 | 5.046E-04 | 3.690E-02 |
| 58 | RP11-37B2.1 | -0.9539 | 0.2948 | 3.2362 | 1.211E-03 | 6.306E-02 |
| 59 | NBR2 | -0.9534 | 0.3023 | 3.154 | 1.611E-03 | 7.436E-02 |
| 60 | CCNL1 | -0.9507 | 0.2167 | 4.3871 | 1.150E-05 | 3.802E-03 |
| 61 | RP6-99M1.2 | -0.9496 | 0.2617 | 3.6279 | 2.857E-04 | 2.493E-02 |
| 62 | MST1L | -0.9437 | 0.3648 | 2.5866 | 9.693E-03 | 1.990E-01 |
| 63 | KLF4 | -0.9419 | 0.3736 | 2.5214 | 1.169E-02 | 2.187E-01 |
| 64 | RP1-179N16.6 | -0.9406 | 0.3052 | 3.0816 | 2.059E-03 | 8.511E-02 |
| 65 | SIAH1 | -0.9354 | 0.3075 | 3.0423 | 2.348E-03 | 9.075E-02 |
| 66 | SLC2A11 | -0.9337 | 0.3154 | 2.9606 | 3.070E-03 | 1.038E-01 |
| 67 | LTB4R | -0.9164 | 0.1596 | 5.7419 | 9.360E-09 | 1.970E-05 |
| 68 | GABBR1 | -0.9153 | 0.2486 | 3.6823 | 2.312E-04 | 2.222E-02 |
| 69 | AC003104.1 | -0.9145 | 0.3059 | 2.9899 | 2.791E-03 | 9.911E-02 |
| 70 | CTC-428G20.3 | -0.9133 | 0.3111 | 2.9355 | 3.330E-03 | 1.069E-01 |
| 71 | RP11-296I10.6 | -0.9105 | 0.316 | 2.8814 | 3.959E-03 | 1.175E-01 |
| 72 | DNAH17-AS1 | -0.9049 | 0.2525 | 3.5831 | 3.396E-04 | 2.883E-02 |
| 73 | CTD-3092A11.2 | -0.9038 | 0.2611 | 3.4614 | 5.373E-04 | 3.797E-02 |
| 74 | WDPCP | -0.8993 | 0.2424 | 3.7104 | 2.070E-04 | 2.111E-02 |
| 75 | KLHL3 | -0.898 | 0.3391 | 2.648 | 8.098E-03 | 1.794E-01 |
| 76 | RP11-564D11.3 | -0.8897 | 0.3147 | 2.8273 | 4.694E-03 | 1.271E-01 |
| 77 | PPIG | -0.8861 | 0.2638 | 3.3588 | 7.828E-04 | 4.761E-02 |
| 78 | LUC7L3 | -0.8856 | 0.2413 | 3.6694 | 2.431E-04 | 2.276E-02 |
| 79 | PIBF1 | -0.8856 | 0.2811 | 3.1499 | 1.633E-03 | 7.459E-02 |
| 80 | CLGN | -0.8853 | 0.2154 | 4.1094 | 3.970E-05 | 8.070E-03 |
| 81 | LRRIQ1 | -0.8837 | 0.3227 | 2.7386 | 6.169E-03 | 1.524E-01 |
| 82 | AC034220.3 | -0.8812 | 0.2922 | 3.0164 | 2.558E-03 | 9.435E-02 |
| 83 | PYROXD1 | -0.8793 | 0.329 | 2.6727 | 7.524E-03 | 1.717E-01 |
| 84 | SLMO1 | -0.8777 | 0.3225 | 2.7212 | 6.505E-03 | 1.567E-01 |
| 85 | RP11-69E11.4 | -0.8777 | 0.3493 | 2.5128 | 1.198E-02 | 2.200E-01 |
| 86 | AP001065.2 | -0.8733 | 0.2783 | 3.1379 | 1.702E-03 | 7.618E-02 |
| 87 | CLDN1 | -0.8729 | 0.2561 | 3.4079 | 6.546E-04 | 4.268E-02 |
| 88 | AKAP9 | -0.8712 | 0.1859 | 4.6856 | 2.790E-06 | 1.961E-03 |
| 89 | SH3BP5-AS1 | -0.8669 | 0.2701 | 3.2094 | 1.330E-03 | 6.651E-02 |
| 90 | RP11-285F7.2 | -0.8656 | 0.3202 | 2.7029 | 6.874E-03 | 1.612E-01 |
| 91 | RP11-819M15.1 | -0.8596 | 0.3437 | 2.5007 | 1.239E-02 | 2.233E-01 |
| 92 | SLC22A15 | -0.8576 | 0.2455 | 3.4932 | 4.772E-04 | 3.593E-02 |
| 93 | TSACC | -0.8546 | 0.3399 | 2.5142 | 1.193E-02 | 2.200E-01 |
| 94 | CNTRL | -0.8534 | 0.2209 | 3.863 | 1.120E-04 | 1.431E-02 |
| 95 | ZNF83 | -0.8529 | 0.2981 | 2.8614 | 4.218E-03 | 1.230E-01 |
| 96 | TUBE1 | -0.852 | 0.1847 | 4.6137 | 3.960E-06 | 2.377E-03 |
| 97 | CEP152 | -0.8481 | 0.2203 | 3.8497 | 1.183E-04 | 1.481E-02 |
| 98 | ZNF204P | -0.8446 | 0.2413 | 3.5004 | 4.646E-04 | 3.540E-02 |
| 99 | KIF18A | -0.8437 | 0.1694 | 4.98 | 6.360E-07 | 7.310E-04 |
| 100 | CCPG1 | -0.8418 | 0.1913 | 4.4008 | 1.080E-05 | 3.790E-03 |
| 101 | C5orf28 | -0.8408 | 0.3424 | 2.4553 | 1.408E-02 | 2.380E-01 |
| 102 | CEP135 | -0.8378 | 0.2134 | 3.9265 | 8.620E-05 | 1.234E-02 |
| 103 | DUSP8 | -0.8364 | 0.3234 | 2.5861 | 9.708E-03 | 1.990E-01 |
| 104 | PNISR | -0.8361 | 0.224 | 3.7334 | 1.889E-04 | 2.025E-02 |
| 105 | CTC-444N24.11 | -0.8343 | 0.2001 | 4.1688 | 3.060E-05 | 6.677E-03 |
| 106 | NCF2 | -0.831 | 0.2877 | 2.8885 | 3.870E-03 | 1.168E-01 |
| 107 | IFRD1 | -0.8293 | 0.2262 | 3.6659 | 2.465E-04 | 2.276E-02 |
| 108 | SRP14-AS1 | -0.829 | 0.2689 | 3.0831 | 2.049E-03 | 8.497E-02 |
| 109 | C4orf21 | -0.8288 | 0.2743 | 3.0216 | 2.515E-03 | 9.411E-02 |
| 110 | PPWD1 | -0.8242 | 0.2439 | 3.3795 | 7.261E-04 | 4.547E-02 |
| 111 | PSAT1 | -0.8237 | 0.3151 | 2.6146 | 8.934E-03 | 1.880E-01 |
| 112 | GOLGA8A | -0.8229 | 0.1973 | 4.1708 | 3.040E-05 | 6.677E-03 |
| 113 | NPIPA1 | -0.8211 | 0.3109 | 2.6415 | 8.253E-03 | 1.813E-01 |
| 114 | MAT2A | -0.8191 | 0.3261 | 2.5117 | 1.202E-02 | 2.200E-01 |
| 115 | SLC25A27 | -0.8165 | 0.2996 | 2.725 | 6.430E-03 | 1.558E-01 |
| 116 | CCDC18 | -0.8162 | 0.2245 | 3.6351 | 2.779E-04 | 2.451E-02 |
| 117 | CHIC1 | -0.8114 | 0.2063 | 3.933 | 8.390E-05 | 1.234E-02 |
| 118 | NFIL3 | -0.8062 | 0.2811 | 2.8679 | 4.131E-03 | 1.213E-01 |
| 119 | CREG2 | -0.805 | 0.1901 | 4.2353 | 2.280E-05 | 5.777E-03 |
| 120 | RP11-1023L17.1 | -0.7971 | 0.315 | 2.5304 | 1.139E-02 | 2.158E-01 |
| 121 | GARS | -0.7921 | 0.2712 | 2.9211 | 3.488E-03 | 1.101E-01 |
| 122 | RP11-420A23.1 | -0.7918 | 0.2917 | 2.7141 | 6.646E-03 | 1.579E-01 |
| 123 | KIAA1551 | -0.7893 | 0.2678 | 2.9474 | 3.204E-03 | 1.056E-01 |
| 124 | PPP1R15B | -0.7876 | 0.2138 | 3.6845 | 2.291E-04 | 2.222E-02 |
| 125 | STRADA | -0.7814 | 0.2844 | 2.7479 | 5.997E-03 | 1.496E-01 |
| 126 | TCEA1 | -0.7809 | 0.2095 | 3.7279 | 1.931E-04 | 2.052E-02 |
| 127 | RP11-700P18.1 | -0.7747 | 0.2901 | 2.6705 | 7.574E-03 | 1.720E-01 |
| 128 | PNN | -0.7743 | 0.2082 | 3.7183 | 2.006E-04 | 2.097E-02 |
| 129 | RP1-93I3.1 | -0.773 | 0.3043 | 2.5406 | 1.107E-02 | 2.113E-01 |
| 130 | ZNF146 | -0.7706 | 0.1735 | 4.4414 | 8.940E-06 | 3.427E-03 |
| 131 | GADD45A | -0.7698 | 0.234 | 3.2904 | 1.000E-03 | 5.600E-02 |
| 132 | RP3-467N11.1 | -0.7683 | 0.3014 | 2.5494 | 1.079E-02 | 2.100E-01 |
| 133 | BMP2 | -0.7672 | 0.2206 | 3.4783 | 5.047E-04 | 3.690E-02 |
| 134 | RP11-890B15.3 | -0.7657 | 0.2706 | 2.8295 | 4.662E-03 | 1.269E-01 |
| 135 | GCC2 | -0.7627 | 0.2099 | 3.634 | 2.790E-04 | 2.451E-02 |
| 136 | SEC63P1 | -0.7621 | 0.2224 | 3.4261 | 6.123E-04 | 4.120E-02 |
| 137 | NEBL | -0.7621 | 0.2527 | 3.0153 | 2.567E-03 | 9.440E-02 |
| 138 | GOLGA8B | -0.7607 | 0.2878 | 2.6428 | 8.222E-03 | 1.811E-01 |
| 139 | RP4-717I23.3 | -0.7577 | 0.2245 | 3.3751 | 7.379E-04 | 4.575E-02 |
| 140 | ATG16L2 | -0.7543 | 0.215 | 3.5087 | 4.502E-04 | 3.516E-02 |
| 141 | THAP9-AS1 | -0.754 | 0.1738 | 4.3391 | 1.430E-05 | 4.525E-03 |
| 142 | PAQR6 | -0.754 | 0.2731 | 2.7606 | 5.769E-03 | 1.463E-01 |
| 143 | ANKRD36 | -0.7538 | 0.2484 | 3.0348 | 2.407E-03 | 9.206E-02 |
| 144 | FAM129A | -0.7486 | 0.2487 | 3.0096 | 2.616E-03 | 9.563E-02 |
| 145 | SYNE1 | -0.7387 | 0.2483 | 2.9744 | 2.936E-03 | 1.015E-01 |
| 146 | DEPDC7 | -0.7347 | 0.2574 | 2.8538 | 4.320E-03 | 1.248E-01 |
| 147 | CHD1 | -0.7338 | 0.1976 | 3.7142 | 2.039E-04 | 2.101E-02 |
| 148 | ZNF25 | -0.732 | 0.2399 | 3.0512 | 2.280E-03 | 9.040E-02 |
| 149 | ATAD5 | -0.731 | 0.2459 | 2.9727 | 2.952E-03 | 1.015E-01 |
| 150 | EIF2S2 | -0.7305 | 0.2908 | 2.5119 | 1.201E-02 | 2.200E-01 |
| 151 | CEP95 | -0.7292 | 0.1918 | 3.801 | 1.441E-04 | 1.642E-02 |
| 152 | C5orf45 | -0.7277 | 0.2388 | 3.0476 | 2.307E-03 | 9.064E-02 |
| 153 | EIF2S2P4 | -0.7253 | 0.2736 | 2.651 | 8.025E-03 | 1.794E-01 |
| 154 | THOC1 | -0.7234 | 0.2723 | 2.6562 | 7.903E-03 | 1.773E-01 |
| 155 | CENPU | -0.7211 | 0.2115 | 3.4102 | 6.492E-04 | 4.255E-02 |
| 156 | RHOBTB3 | -0.7191 | 0.2678 | 2.6849 | 7.254E-03 | 1.672E-01 |
| 157 | SRFBP1 | -0.7166 | 0.256 | 2.7991 | 5.125E-03 | 1.342E-01 |
| 158 | MFI2-AS1 | -0.7161 | 0.2917 | 2.4549 | 1.409E-02 | 2.380E-01 |
| 159 | GGNBP2 | -0.7156 | 0.1823 | 3.9248 | 8.680E-05 | 1.234E-02 |
| 160 | PDCD4 | -0.7154 | 0.2701 | 2.6485 | 8.086E-03 | 1.794E-01 |
| 161 | FAM133B | -0.7153 | 0.2421 | 2.9551 | 3.126E-03 | 1.047E-01 |
| 162 | ATP6AP1L | -0.7087 | 0.2379 | 2.9793 | 2.889E-03 | 1.007E-01 |
| 163 | ZNF84 | -0.7058 | 0.22 | 3.2081 | 1.336E-03 | 6.653E-02 |
| 164 | RIOK3 | -0.704 | 0.2124 | 3.3144 | 9.183E-04 | 5.353E-02 |
| 165 | CPNE7 | -0.7036 | 0.1921 | 3.6632 | 2.491E-04 | 2.284E-02 |
| 166 | UPF3B | -0.703 | 0.2478 | 2.8376 | 4.545E-03 | 1.260E-01 |
| 167 | PLCB4 | -0.7002 | 0.1897 | 3.6911 | 2.233E-04 | 2.218E-02 |
| 168 | RPGR | -0.6987 | 0.1897 | 3.6827 | 2.308E-04 | 2.222E-02 |
| 169 | UACA | -0.6951 | 0.205 | 3.3913 | 6.957E-04 | 4.387E-02 |
| 170 | CCDC66 | -0.6947 | 0.228 | 3.0476 | 2.307E-03 | 9.064E-02 |
| 171 | JMJD1C | -0.6943 | 0.1532 | 4.5327 | 5.820E-06 | 2.540E-03 |
| 172 | C1orf27 | -0.6913 | 0.1987 | 3.4786 | 5.041E-04 | 3.690E-02 |
| 173 | AC132872.2 | -0.6901 | 0.2518 | 2.7403 | 6.139E-03 | 1.520E-01 |
| 174 | CCDC14 | -0.6876 | 0.1953 | 3.5216 | 4.290E-04 | 3.434E-02 |
| 175 | KTN1 | -0.6866 | 0.2536 | 2.7072 | 6.785E-03 | 1.595E-01 |
| 176 | PHLDB2 | -0.6851 | 0.2014 | 3.4012 | 6.710E-04 | 4.353E-02 |
| 177 | AC019097.7 | -0.6817 | 0.2407 | 2.832 | 4.626E-03 | 1.264E-01 |
| 178 | CEP120 | -0.6797 | 0.187 | 3.6352 | 2.778E-04 | 2.451E-02 |
| 179 | SRSF5 | -0.6794 | 0.2346 | 2.8956 | 3.785E-03 | 1.160E-01 |
| 180 | MAPK6 | -0.678 | 0.1487 | 4.56 | 5.110E-06 | 2.377E-03 |
| 181 | CASP8AP2 | -0.677 | 0.2634 | 2.5704 | 1.016E-02 | 2.036E-01 |
| 182 | MSH5 | -0.6763 | 0.2077 | 3.2559 | 1.130E-03 | 6.046E-02 |
| 183 | RBAK | -0.6751 | 0.1945 | 3.4703 | 5.199E-04 | 3.758E-02 |
| 184 | CSGALNACT2 | -0.675 | 0.2014 | 3.3516 | 8.033E-04 | 4.862E-02 |
| 185 | RPL36A | -0.6747 | 0.2726 | 2.4749 | 1.333E-02 | 2.308E-01 |
| 186 | OLR1 | -0.6746 | 0.2459 | 2.7436 | 6.077E-03 | 1.510E-01 |
| 187 | CEP85L | -0.6739 | 0.2433 | 2.7698 | 5.609E-03 | 1.434E-01 |
| 188 | IDH1 | -0.6733 | 0.161 | 4.1825 | 2.880E-05 | 6.677E-03 |
| 189 | BEND6 | -0.673 | 0.2713 | 2.4808 | 1.311E-02 | 2.300E-01 |
| 190 | GATAD1 | -0.6697 | 0.1731 | 3.8684 | 1.096E-04 | 1.414E-02 |
| 191 | PLK1S1 | -0.6646 | 0.2462 | 2.6997 | 6.941E-03 | 1.612E-01 |
| 192 | ZRANB2 | -0.6645 | 0.1945 | 3.4158 | 6.360E-04 | 4.215E-02 |
| 193 | ANK2 | -0.6621 | 0.2343 | 2.8254 | 4.723E-03 | 1.274E-01 |
| 194 | NFE2L2 | -0.6607 | 0.1725 | 3.8296 | 1.284E-04 | 1.561E-02 |
| 195 | ADAM33 | -0.6599 | 0.2663 | 2.4782 | 1.320E-02 | 2.307E-01 |
| 196 | RP11-395P17.3 | -0.6552 | 0.2411 | 2.7171 | 6.586E-03 | 1.579E-01 |
| 197 | CIR1 | -0.6541 | 0.1596 | 4.0976 | 4.170E-05 | 8.070E-03 |
| 198 | KIAA1407 | -0.654 | 0.2583 | 2.5322 | 1.133E-02 | 2.149E-01 |
| 199 | MTHFD2 | -0.6537 | 0.2437 | 2.6821 | 7.316E-03 | 1.682E-01 |
| 200 | DNAJC2 | -0.6533 | 0.2419 | 2.7004 | 6.925E-03 | 1.612E-01 |
| 201 | KIAA1731 | -0.6525 | 0.222 | 2.9387 | 3.296E-03 | 1.063E-01 |
| 202 | C9orf85 | -0.6521 | 0.1831 | 3.5617 | 3.684E-04 | 3.087E-02 |
| 203 | ZFAND1 | -0.6511 | 0.179 | 3.6379 | 2.748E-04 | 2.451E-02 |
| 204 | TTC37 | -0.651 | 0.1812 | 3.5919 | 3.283E-04 | 2.824E-02 |
| 205 | FAM206A | -0.6507 | 0.2038 | 3.1928 | 1.409E-03 | 6.864E-02 |
| 206 | XPOT | -0.6502 | 0.2606 | 2.4949 | 1.260E-02 | 2.248E-01 |
| 207 | RBM41 | -0.6499 | 0.2137 | 3.0416 | 2.353E-03 | 9.075E-02 |
| 208 | AKTIP | -0.6482 | 0.2142 | 3.0255 | 2.482E-03 | 9.373E-02 |
| 209 | KIAA0020 | -0.6448 | 0.2264 | 2.8481 | 4.399E-03 | 1.251E-01 |
| 210 | ZMYM5 | -0.6433 | 0.2601 | 2.4737 | 1.337E-02 | 2.308E-01 |
| 211 | TRAPPC6B | -0.6419 | 0.2185 | 2.9382 | 3.301E-03 | 1.063E-01 |
| 212 | KIAA1377 | -0.6399 | 0.2109 | 3.0346 | 2.409E-03 | 9.206E-02 |
| 213 | CTB-43E15.3 | -0.6362 | 0.2546 | 2.4991 | 1.245E-02 | 2.233E-01 |
| 214 | ARGLU1 | -0.6336 | 0.2262 | 2.8013 | 5.090E-03 | 1.336E-01 |
| 215 | RP11-258C19.7 | -0.6334 | 0.2478 | 2.5562 | 1.058E-02 | 2.079E-01 |
| 216 | MBD4 | -0.6292 | 0.2186 | 2.8787 | 3.993E-03 | 1.180E-01 |
| 217 | PHF3 | -0.6289 | 0.2383 | 2.6389 | 8.317E-03 | 1.820E-01 |
| 218 | PKD1P6 | -0.6285 | 0.2577 | 2.4393 | 1.472E-02 | 2.457E-01 |
| 219 | IGFBP3 | -0.6258 | 0.2256 | 2.7744 | 5.531E-03 | 1.419E-01 |
| 220 | ZC3H8 | -0.6228 | 0.2443 | 2.5488 | 1.081E-02 | 2.100E-01 |
| 221 | CPEB4 | -0.6197 | 0.169 | 3.6679 | 2.445E-04 | 2.276E-02 |
| 222 | UFSP2 | -0.6186 | 0.214 | 2.8913 | 3.837E-03 | 1.167E-01 |
| 223 | NR1D2 | -0.6183 | 0.2249 | 2.7495 | 5.969E-03 | 1.493E-01 |
| 224 | CLIC4 | -0.6179 | 0.1995 | 3.0976 | 1.951E-03 | 8.309E-02 |
| 225 | CWC27 | -0.6165 | 0.2329 | 2.6471 | 8.118E-03 | 1.795E-01 |
| 226 | ZCCHC7 | -0.6138 | 0.1751 | 3.5063 | 4.544E-04 | 3.527E-02 |
| 227 | MAP3K2 | -0.6121 | 0.2377 | 2.5754 | 1.001E-02 | 2.017E-01 |
| 228 | TSEN15 | -0.6095 | 0.2108 | 2.8913 | 3.836E-03 | 1.167E-01 |
| 229 | SEC63 | -0.6081 | 0.1486 | 4.0915 | 4.290E-05 | 8.070E-03 |
| 230 | AZI2 | -0.6042 | 0.2292 | 2.6357 | 8.397E-03 | 1.830E-01 |
| 231 | NARS | -0.6036 | 0.2415 | 2.4997 | 1.243E-02 | 2.233E-01 |
| 232 | ARMC4 | -0.6019 | 0.1887 | 3.189 | 1.428E-03 | 6.864E-02 |
| 233 | NHS | -0.6015 | 0.2077 | 2.8954 | 3.787E-03 | 1.160E-01 |
| 234 | PTPLB | -0.5996 | 0.2462 | 2.4351 | 1.489E-02 | 2.468E-01 |
| 235 | EZH1 | -0.5992 | 0.1809 | 3.3126 | 9.243E-04 | 5.363E-02 |
| 236 | CTH | -0.5982 | 0.2105 | 2.842 | 4.483E-03 | 1.258E-01 |
| 237 | TCEA1P2 | -0.5958 | 0.1816 | 3.281 | 1.034E-03 | 5.662E-02 |
| 238 | POLI | -0.5955 | 0.2366 | 2.5165 | 1.185E-02 | 2.196E-01 |
| 239 | FGF2 | -0.5954 | 0.1814 | 3.2825 | 1.029E-03 | 5.659E-02 |
| 240 | NCOA7 | -0.5949 | 0.1806 | 3.2936 | 9.891E-04 | 5.561E-02 |
| 241 | SIRT1 | -0.5906 | 0.1715 | 3.4442 | 5.728E-04 | 3.896E-02 |
| 242 | LENG8 | -0.5901 | 0.21 | 2.8095 | 4.961E-03 | 1.316E-01 |
| 243 | APIP | -0.5871 | 0.2163 | 2.7144 | 6.641E-03 | 1.579E-01 |
| 244 | BACH1 | -0.5864 | 0.17 | 3.4487 | 5.633E-04 | 3.896E-02 |
| 245 | EPRS | -0.586 | 0.2372 | 2.4701 | 1.351E-02 | 2.324E-01 |
| 246 | CKAP2L | -0.5857 | 0.2194 | 2.6688 | 7.612E-03 | 1.723E-01 |
| 247 | JMY | -0.5848 | 0.1952 | 2.995 | 2.744E-03 | 9.806E-02 |
| 248 | ZNF182 | -0.5837 | 0.239 | 2.4418 | 1.461E-02 | 2.445E-01 |
| 249 | AHSA2 | -0.5835 | 0.2144 | 2.7213 | 6.503E-03 | 1.567E-01 |
| 250 | SKIL | -0.5825 | 0.2259 | 2.5783 | 9.929E-03 | 2.013E-01 |
| 251 | PLD1 | -0.5803 | 0.2027 | 2.8635 | 4.190E-03 | 1.224E-01 |
| 252 | USP15 | -0.5797 | 0.2381 | 2.4346 | 1.491E-02 | 2.468E-01 |
| 253 | SYF2 | -0.5781 | 0.2018 | 2.8646 | 4.175E-03 | 1.223E-01 |
| 254 | LYST | -0.5751 | 0.166 | 3.4641 | 5.320E-04 | 3.797E-02 |
| 255 | ZNF33A | -0.5749 | 0.2293 | 2.5074 | 1.216E-02 | 2.218E-01 |
| 256 | ZMYM6 | -0.5739 | 0.2015 | 2.8485 | 4.393E-03 | 1.251E-01 |
| 257 | TOP2A | -0.5726 | 0.2316 | 2.4721 | 1.343E-02 | 2.315E-01 |
| 258 | DCUN1D4 | -0.5703 | 0.1976 | 2.8864 | 3.896E-03 | 1.168E-01 |
| 259 | HAUS6 | -0.5694 | 0.192 | 2.9656 | 3.021E-03 | 1.027E-01 |
| 260 | CAPRIN2 | -0.5693 | 0.2036 | 2.7958 | 5.178E-03 | 1.351E-01 |
| 261 | LINC00669 | -0.5691 | 0.2034 | 2.7983 | 5.137E-03 | 1.343E-01 |
| 262 | PVT1 | -0.5675 | 0.162 | 3.5039 | 4.584E-04 | 3.536E-02 |
| 263 | RBMS1 | -0.5673 | 0.201 | 2.8226 | 4.764E-03 | 1.277E-01 |
| 264 | TMTC2 | -0.5669 | 0.2289 | 2.4763 | 1.328E-02 | 2.308E-01 |
| 265 | LINC00473 | -0.5665 | 0.1847 | 3.0672 | 2.161E-03 | 8.817E-02 |
| 266 | NCK1 | -0.5655 | 0.2248 | 2.5154 | 1.189E-02 | 2.196E-01 |
| 267 | TMEM242 | -0.5632 | 0.1818 | 3.0977 | 1.951E-03 | 8.309E-02 |
| 268 | ZNF675 | -0.5602 | 0.2027 | 2.7633 | 5.722E-03 | 1.454E-01 |
| 269 | ZC3H15 | -0.5599 | 0.1784 | 3.1384 | 1.699E-03 | 7.618E-02 |
| 270 | ZEB1 | -0.559 | 0.2299 | 2.4315 | 1.504E-02 | 2.478E-01 |
| 271 | ZRSR2 | -0.5575 | 0.2186 | 2.5501 | 1.077E-02 | 2.100E-01 |
| 272 | ORMDL1 | -0.5534 | 0.225 | 2.4592 | 1.393E-02 | 2.365E-01 |
| 273 | CWF19L2 | -0.5514 | 0.189 | 2.9178 | 3.525E-03 | 1.109E-01 |
| 274 | ANXA3 | -0.5503 | 0.1961 | 2.8067 | 5.005E-03 | 1.322E-01 |
| 275 | RP11-22B23.1 | -0.5494 | 0.2112 | 2.6016 | 9.279E-03 | 1.927E-01 |
| 276 | NAA38 | -0.5463 | 0.1962 | 2.7848 | 5.356E-03 | 1.383E-01 |
| 277 | ZNF605 | -0.5459 | 0.181 | 3.0167 | 2.556E-03 | 9.435E-02 |
| 278 | SENP7 | -0.5457 | 0.2205 | 2.4748 | 1.333E-02 | 2.308E-01 |
| 279 | N4BP2L2 | -0.5432 | 0.1692 | 3.2113 | 1.321E-03 | 6.651E-02 |
| 280 | KIAA1456 | -0.5412 | 0.2116 | 2.5574 | 1.054E-02 | 2.074E-01 |
| 281 | CCDC59 | -0.5352 | 0.2104 | 2.5431 | 1.099E-02 | 2.109E-01 |
| 282 | RNF217 | -0.5344 | 0.1602 | 3.3351 | 8.526E-04 | 5.064E-02 |
| 283 | BIRC2 | -0.5344 | 0.1617 | 3.3037 | 9.541E-04 | 5.412E-02 |
| 284 | HINT3 | -0.5333 | 0.1664 | 3.2058 | 1.347E-03 | 6.683E-02 |
| 285 | KIF3A | -0.5324 | 0.2001 | 2.661 | 7.791E-03 | 1.757E-01 |
| 286 | PAWR | -0.5306 | 0.1701 | 3.1202 | 1.807E-03 | 7.939E-02 |
| 287 | TMEM168 | -0.5294 | 0.1948 | 2.7181 | 6.567E-03 | 1.579E-01 |
| 288 | SCFD1 | -0.529 | 0.1961 | 2.6985 | 6.966E-03 | 1.614E-01 |
| 289 | TTC28-AS1 | -0.5289 | 0.1953 | 2.7087 | 6.755E-03 | 1.594E-01 |
| 290 | NIFK | -0.5278 | 0.206 | 2.5624 | 1.039E-02 | 2.058E-01 |
| 291 | C4orf33 | -0.5254 | 0.2068 | 2.5403 | 1.108E-02 | 2.113E-01 |
| 292 | DAAM1 | -0.5238 | 0.1808 | 2.8974 | 3.763E-03 | 1.158E-01 |
| 293 | SMIM20 | -0.522 | 0.1995 | 2.6171 | 8.868E-03 | 1.875E-01 |
| 294 | CEP57 | -0.5215 | 0.1704 | 3.061 | 2.206E-03 | 8.830E-02 |
| 295 | ANXA1 | -0.5211 | 0.1545 | 3.3715 | 7.475E-04 | 4.613E-02 |
| 296 | DST | -0.5173 | 0.2036 | 2.5413 | 1.104E-02 | 2.113E-01 |
| 297 | CREG1 | -0.5152 | 0.1638 | 3.1454 | 1.659E-03 | 7.548E-02 |
| 298 | GS1-251I9.4 | -0.5087 | 0.2014 | 2.5262 | 1.153E-02 | 2.174E-01 |
| 299 | PAPD4 | -0.5086 | 0.1589 | 3.2004 | 1.372E-03 | 6.755E-02 |
| 300 | PABPN1 | -0.5086 | 0.1629 | 3.1226 | 1.792E-03 | 7.928E-02 |
| 301 | FIP1L1 | -0.5056 | 0.1734 | 2.9151 | 3.556E-03 | 1.116E-01 |
| 302 | ARL5B | -0.4969 | 0.2002 | 2.4828 | 1.304E-02 | 2.293E-01 |
| 303 | RSL24D1 | -0.4969 | 0.2047 | 2.4271 | 1.522E-02 | 2.491E-01 |
| 304 | UFM1 | -0.4935 | 0.184 | 2.6817 | 7.325E-03 | 1.682E-01 |
| 305 | MCC | -0.4932 | 0.203 | 2.4295 | 1.512E-02 | 2.484E-01 |
| 306 | LUC7L | -0.4908 | 0.1668 | 2.942 | 3.261E-03 | 1.063E-01 |
| 307 | SLC25A16 | -0.4891 | 0.1953 | 2.5045 | 1.226E-02 | 2.232E-01 |
| 308 | DDX21 | -0.4881 | 0.1476 | 3.3067 | 9.441E-04 | 5.404E-02 |
| 309 | PLEKHA5 | -0.4865 | 0.1947 | 2.4987 | 1.247E-02 | 2.233E-01 |
| 310 | IFNGR1 | -0.4841 | 0.1701 | 2.8456 | 4.433E-03 | 1.251E-01 |
| 311 | DNAH14 | -0.4776 | 0.1677 | 2.8473 | 4.409E-03 | 1.251E-01 |
| 312 | RSRC1 | -0.4743 | 0.1724 | 2.7505 | 5.951E-03 | 1.493E-01 |
| 313 | ATAD1 | -0.4705 | 0.1413 | 3.3308 | 8.658E-04 | 5.078E-02 |
| 314 | PTPN12 | -0.4683 | 0.1711 | 2.7371 | 6.199E-03 | 1.526E-01 |
| 315 | PCID2 | -0.4678 | 0.167 | 2.8012 | 5.092E-03 | 1.336E-01 |
| 316 | DONSON | -0.4659 | 0.1341 | 3.4747 | 5.113E-04 | 3.717E-02 |
| 317 | CHURC1 | -0.4569 | 0.1492 | 3.062 | 2.199E-03 | 8.830E-02 |
| 318 | SNX24 | -0.4556 | 0.1822 | 2.4999 | 1.242E-02 | 2.233E-01 |
| 319 | HNRNPH3 | -0.4555 | 0.1355 | 3.3618 | 7.744E-04 | 4.732E-02 |
| 320 | ING5 | -0.4544 | 0.166 | 2.7376 | 6.190E-03 | 1.526E-01 |
| 321 | TRMT11 | -0.4528 | 0.1681 | 2.6935 | 7.070E-03 | 1.635E-01 |
| 322 | C16orf87 | -0.4521 | 0.1808 | 2.5002 | 1.241E-02 | 2.233E-01 |
| 323 | TANK | -0.4519 | 0.1826 | 2.4744 | 1.335E-02 | 2.308E-01 |
| 324 | RC3H1 | -0.4467 | 0.1812 | 2.4646 | 1.372E-02 | 2.346E-01 |
| 325 | IFT81 | -0.4459 | 0.163 | 2.7356 | 6.226E-03 | 1.528E-01 |
| 326 | NR4A2 | -0.4442 | 0.1509 | 2.9435 | 3.245E-03 | 1.063E-01 |
| 327 | FBXO5 | -0.4349 | 0.1632 | 2.6647 | 7.706E-03 | 1.741E-01 |
| 328 | RBM39 | -0.4324 | 0.1438 | 3.0066 | 2.642E-03 | 9.603E-02 |
| 329 | SDCCAG8 | -0.4198 | 0.17 | 2.4697 | 1.352E-02 | 2.324E-01 |
| 330 | MARK3 | -0.4132 | 0.1525 | 2.7091 | 6.747E-03 | 1.594E-01 |
| 331 | SLMO2 | -0.4088 | 0.1368 | 2.9892 | 2.797E-03 | 9.911E-02 |
| 332 | EME1 | -0.4084 | 0.1552 | 2.6311 | 8.512E-03 | 1.834E-01 |
| 333 | SLC25A36 | -0.4049 | 0.1601 | 2.5294 | 1.143E-02 | 2.160E-01 |
| 334 | PTPDC1 | -0.4009 | 0.1602 | 2.502 | 1.235E-02 | 2.233E-01 |
| 335 | RFC4 | -0.3927 | 0.1576 | 2.4922 | 1.270E-02 | 2.259E-01 |
| 336 | MTAP | -0.3912 | 0.143 | 2.7353 | 6.233E-03 | 1.528E-01 |
| 337 | CHEK1 | -0.3911 | 0.1476 | 2.6487 | 8.080E-03 | 1.794E-01 |
| 338 | EIF4G3 | -0.3903 | 0.1417 | 2.7547 | 5.874E-03 | 1.485E-01 |
| 339 | QRSL1 | -0.3722 | 0.1451 | 2.5644 | 1.034E-02 | 2.056E-01 |
| 340 | DCAF16 | -0.3698 | 0.1521 | 2.4312 | 1.505E-02 | 2.478E-01 |
| 341 | CDC42SE2 | -0.3583 | 0.1476 | 2.428 | 1.518E-02 | 2.488E-01 |
| 342 | NUB1 | -0.3556 | 0.1309 | 2.7164 | 6.599E-03 | 1.579E-01 |

**Table S3.** Clinicopathological characteristics of HCC patients (n=45)

| Characteristic | Cases | % |
| --- | --- | --- |
| Age (years) |  |  |
| <60 | 31 | 68.9 |
| ≥60 | 14 | 31.1 |
| Gender |  |  |
| male | 38 | 84.4 |
| female | 7 | 15.6 |
| pTNM stage |  |  |
| I/II | 19 | 42.2 |
| III | 26 | 57.8 |
| Tumor size |  |  |
| <5 cm | 16 | 35.6 |
| ≥5 cm | 29 | 64.4 |


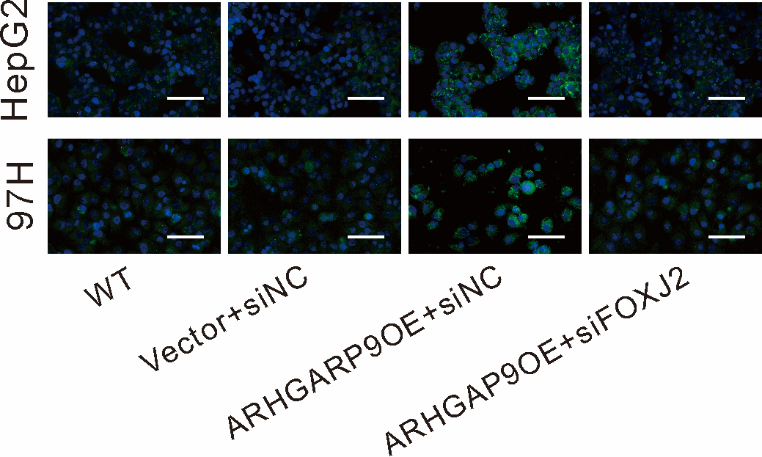


**Figure S1.** ARHGAP9 enhanced E-cadherin expression via FOXJ2. HepG2 and MHCC-97H cells were divided into 4 groups. At 48h post treatment, the cells were incubated with anti-E-cadherin and then with Alexa Fluor 488-conjugated secondary antibody (Green). DAPI staining identified cell nuclei (Blue). Scale bar: 50 μm.
